# Causal relationships between susceptibility and severity of COVID-19 and neuromyelitis optica spectrum disorder (NMOSD) in European population: a bidirectional Mendelian randomized study

**DOI:** 10.3389/fimmu.2023.1305650

**Published:** 2023-12-04

**Authors:** Shengnan Wang, Lijuan Wang, Jianglong Wang, Mingqin Zhu

**Affiliations:** ^1^ Department of Neurology, Neuroscience Center, The First Hospital of Jilin University, Changchun, China; ^2^ First Operating Room, The First Hospital of Jilin University, Changchun, China

**Keywords:** Mendelian randomization, COVID-19, SARS-CoV-2, NMOSD, genome-wide association study

## Abstract

**Background:**

Neurological disorders can be caused by viral infections. The association between viral infections and neuromyelitis optica spectrum disorder (NMOSD) has been well-documented for a long time, and this connection has recently come to attention with the occurrence of SARS-CoV-2 infection. However, the precise nature of the causal connection between NMOSD and COVID-19 infection remains uncertain.

**Methods:**

To investigate the causal relationship between COVID-19 and NMOSD, we utilized a two-sample Mendelian randomization (MR) approach. This analysis was based on the most extensive and recent genome-wide association study (GWAS) that included SARS-CoV-2 infection data (122616 cases and 2475240 controls), hospitalized COVID-19 data (32519 cases and 2062805 controls), and data on severe respiratory confirmed COVID-19 cases (13769 cases and 1072442 controls). Additionally, we incorporated a GWAS meta-analysis comprising 132 cases of AQP4-IgG-seropositive NMOSD (NMO-IgG+), 83 cases of AQP4-IgG-seronegative NMOSD (NMO-IgG−), and 1244 controls.

**Results:**

The findings of our study indicate that the risk of developing NMO-IgG+ is elevated when there is a genetic predisposition to SARS-CoV-2 infection (OR = 5.512, 95% CI = 1.403-21.657, P = 0.014). Furthermore, patients with genetically predicted NMOSD did not exhibit any heightened susceptibility to SARS-CoV2 infection, COVID-19 hospitalization, or severity.

**Conclusion:**

our study using Mendelian randomization (MR) revealed, for the first time, that the presence of genetically predicted SARS-CoV2 infection was identified as a contributing factor for NMO-IgG+ relapses.

## Introduction

The pandemic caused by the highly contagious respiratory illness known as coronavirus disease 2019 (COVID-19) originated in Wuhan, China in December 2019. On March 11, 2020, the World Health Organization officially declared it a pandemic, caused by the severe acute respiratory syndrome coronavirus 2 (SARS-CoV-2) (1). By January 1, 2023, the global impact of the COVID-19 pandemic has resulted in a devastating death toll exceeding 6 million people, while the number of confirmed cases has surpassed 661 million. From the initial phases of the epidemic, there has been an indication that people with weakened immune systems might have a greater chance of getting infected and facing more severe manifestations of the illness (2).

Neuromyelitis optica spectrum disorder (NMOSD), a rare autoimmune astrocytopathy affecting the central nervous system (CNS), is identified by optic neuritis and longitudinally extensive myelitis longitudinally extensive myelitis (LETM) (3). The existence of autoantibodies targeting aquaporin-4 (AQP4) is a defining characteristic of NMOSD (4). Young adults are the most affected by NMOSD, with a higher prevalence among women (5). Due to the debilitating characteristics of NMOSD and its management with immunosuppressive treatments, individuals with this disorder have a higher susceptibility to acquiring bacterial and viral infections (6).

A comprehensive analysis of numerous epidemiological investigations has revealed that individuals diagnosed with NMOSD face a higher susceptibility to severe COVID-19 because of underlying health conditions and the administration of rituximab (2). Eisler et al. provided evidence indicating that the administration of anti-CD20 therapy to individuals with NMOSD may attenuate the protective efficacy of mRNA-based COVID-19 vaccines (7). Nevertheless, some investigations indicated that the severity of disability in individuals with NMOSD, the particular form of disease-modifying treatment (DMT) employed, and any accompanying medical conditions did not display a significant correlation with the outcomes of COVID-19 (8). As per the study conducted by Fan et al., individuals diagnosed with multiple sclerosis (MS) or NMOSD did not show a higher susceptibility to COVID-19 infection, irrespective of their DMT treatment (9). In a clinical investigation encompassing Turkish patients diagnosed with NMOSD and MOGAD, it was observed that advanced age, elevated disability levels, and the coexistence of comorbid conditions contribute to heightened susceptibility to severe COVID-19 infection. Furthermore, the study did not identify any direct and statistically significant impact of DMTs on the occurrence of COVID-19 infection (10). A prior literature review concluded that the advantages associated with the prevention of further disease progression in multiple sclerosis (MS) or neuromyelitis optica spectrum disorder (NMOSD) are likely to surpass the risk of infection for almost all treatment modalities (11). Variations in these findings could be ascribed to possible confounding variables, the size of the sample, and the heterogeneity of the population. Despite concerns raised by researchers regarding the possible link between COVID-19 and NMOSD, there is currently a lack of precise information on the prevalence of SARS-CoV-2 infection among individuals with NMOSD.

Meanwhile, COVID-19 has been found to be linked with a heightened susceptibility to various neurological and psychiatric consequences (12). These encompass both acute and chronic symptoms, such as anosmia, ageusia, cognitive impairments, depression, and anxiety (13). The presence of neuroinflammation alone can disrupt the regulation of glial and neuronal cells, leading to neural circuit dysfunction and subsequent adverse effects on cognitive and neuropsychiatric functions (14). Thus far, human autopsy investigations have revealed the presence of viral RNA transcripts in cerebral tissues, as well as viral proteins within the endothelial cells located in the olfactory bulb of individuals who have perished due to COVID-19 (15).

Currently, there is limited understanding regarding the mechanisms through which SARS-CoV-2 infection may induce NMOSD. However, the notion that viral infections can serve as triggers for NMOSD pathogenesis is supported by numerous case series and reports, which have demonstrated an association between NMOSD and various viral infections such as Epstein Barr virus, influenza virus, human immunodeficiency viruses (HIV), and varicella zoster virus (16–21). In addition, SARS-CoV-2 infection has emerged as a potential risk factor for both peripheral nervous system (PNS) and CNS demyelinating diseases, aligning it with other viral agents (22). In recent research, the safety and effectiveness of COVID-19 vaccines in individuals with NMOSD have been assessed (23).

Additional research is advantageous in enhancing comprehension of the viability and potential hazards associated with vaccination in individuals with NMOSD. However, the severe detrimental consequences of COVID-19 preclude the feasibility of conducting randomized controlled trials (RCTs), the prevailing method for establishing causality, to investigate the causal association between COVID-19 and these deleterious health outcomes. Consequently, the precise causal relationship between COVID-19 and NMOSD remains elusive. Elucidating the connection between COVID-19 infection and NMOSD is imperative for advancing research pertaining to the diagnosis, treatment, and recuperation of individuals afflicted with NMOSD who have contracted COVID-19. Moreover, it is imperative for formulating effective approaches to manage and provide care for NMOSD patients in the context of a COVID-19 infection.

Mendelian randomization (MR) is an epidemiological technique that employs Single nucleotide polymorphisms (SNPs) as instrumental variables (IVs) to replicate the structure of randomized controlled trials and investigate the causal impacts of a risk factor on a specific outcome.MR analysis is considered more advantageous than observational studies because it helps to mitigate confounding factors and reverse causation (24). Using the bidirectional MR method, we conducted a study to explore the possible causal link between the susceptibility to NMOSD and the occurrence of COVID-19 infection.

## Methods

### Study design

**Figure 1:**
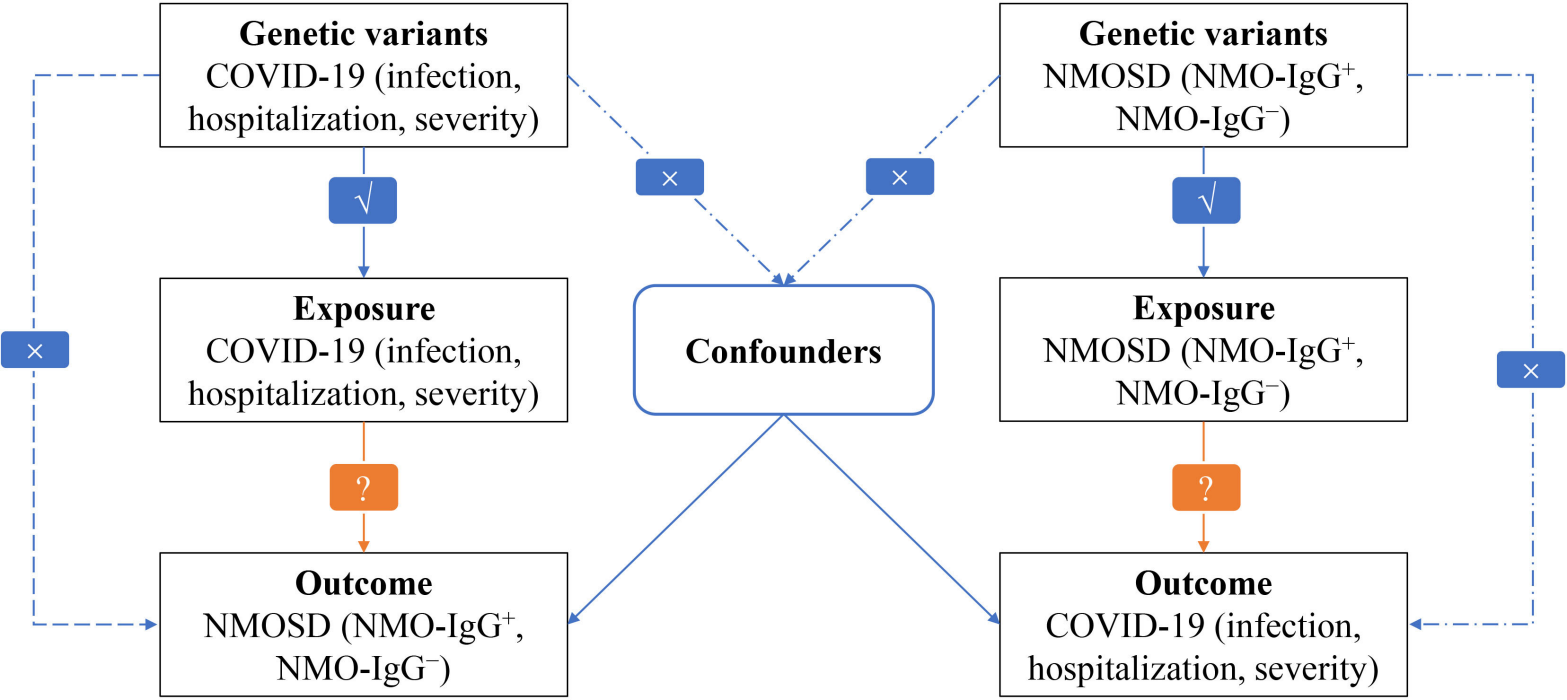
Flowchart of MR analysis in this study. √ : Feasible. × : Not feasible ?: Unknown, waiting to be explored.

In our research, we employed the bidirectional MR method to assess the impact of both COVID-19 on NMOSD and NMOSD on COVID-19 (25). The analysis of MR is based on three important assumptions: (i) there is a significant association between genetic variation and the exposure, (ii) genetic variation is connected to the exposure but not to any confounding factors related to the outcome, and (iii) genetic variation is not linked to the exposure or any outcome dependent on confounding factors (26). **Figure 1** illustrates a visual depiction of the causal connections between the exposure, genetic variation, and outcome variables, offering a summary of the overall MR design employed in our investigation.

### The selection of data sources and instruments

Population stratification can lead to confounding of the gene-disease association due to ethnic factors, particularly when the study population comprises a diverse range of global populations. This confounding by ethnicity may introduce bias in estimating the association between exposure and outcome (27). Consequently, Mendelian randomization studies typically necessitate that both the exposure and outcome variables are of the same racial background. To avoid this problem, we gathered data from European countries of European descent to acquire summary-level genome-wide association study (GWAS) data for genetically predicted COVID-19 risk (binary traits). The GWAS was from the latest version of the COVID-19 Host Genetics Initiative (HGI) GWAS meta-analyzes, round 7 (28). The data consisted of 122616 cases and 2475240 control individuals for SARS-CoV-2 infection, 32519 cases and 2062805 control individuals for hospitalized COVID-19, and 13769 cases and 1072442 control individuals for confirmed COVID-19 with very severe respiratory symptoms. The mean age of the individuals diagnosed with COVID-19 in various studies was found to be 55 years. These individuals willingly participated in the study by accessing a microsite and were obligated to provide self-reported information regarding a positive COVID-19 test. The selection of volunteers for genome sequencing was based on demographic criteria, including self-reported ancestry, gender, age, and location within the United Kingdom, in order to match the critical COVID-19 cohort. Patients who were admitted to the hospital because of laboratory-confirmed SARS-CoV-2 infection and symptoms related to the coronavirus were considered as hospitalized COVID-19 cases. On the other hand, patients who required respiratory support or died because of laboratory-confirmed SARS-CoV-2 infection were classified as very severe respiratory confirmed COVID-19 cases. The individuals were enlisted as participants in the GenOMICC study across 224 intensive care units in the United Kingdom (https://genomicc.org). Individuals with hematological malignancies were excluded to mitigate the risk of potential tumor contamination. Additional information on the COVID-19 GWAS can be found in **Supplementary Tables 1–4**.

We acquired summary-level GWAS data for genetically predicted NMOSD risk, which included 132 cases of AQP4-IgG-seropositive NMOSD, 83 cases of AQP4-IgG-seronegative NMOSD, and 1244 control subjects. Patients were included to form a primary cohort in this study if they fulfilled the criteria established by the International Panel for NMOSD diagnosis in 2015 (29). Our analyses were classified into three categories based on the serum samples: NMO-IgG+ (AQP4-IgG-seropositive NMOSD), NMO-IgG- (AQP4-IgG-seronegative NMOSD), and NMO (combined NMOSD regardless of AQP4-IgG status). All participants were explicitly queried regarding the absence of the subsequent disorders: Alzheimer’s, amyotrophic lateral sclerosis, ataxia, autism, bipolar disorder, cerebrovascular disease, dementia, dystonia, Parkinson’s, and schizophrenia. The characteristics of the NMOSD patients and their clinical presentation are succinctly outlined in **Table 1**. It is crucial to mention that our research solely relied on publicly accessible summary-level information from published meta-analyses of genome-wide association studies and did not include individual or study-level aggregated findings. **Table 2** summarizes the basic characteristics of GWAS, including exposures and outcomes.

**Table 1:** Sample characteristics of NMOSD cases.

	NMO-IgG+		NMO-IgG-	
	Mean	S.D.	Mean	S.D.
Age	48.89	14.83	43.5	11.61
Gender (% Female)	86%		74%	
Type of first symptoms				
Visual	33%		39%	
Spinal	61%		39%	
Both	6%		22%	
Smoker (%)	35%		64%	
Years Diagnosed	2.26	2.19	2.18	2.39
Years Symptoms	5.79	6.12	6.47	7.21
Total C4	3.71	0.73	3.23	0.64

NMO-IgG+, aquaporin 4 IgG seropositive; NMO-IgG-, aquaporin 4 IgG seronegative; S.D., standard deviation.

**Table 2:** The summary data and database number of the GWAS.

Phenotype	Trait contains	ID	Source
COVID-19	Very severe respiratory confirmed COVID-19	–	https://www.covid19hg.org/results/r7/
	Hospitalized COVID-19	–	
	SARS-CoV-2 infection	–	
NMOSD	AQP4-IgG-seropositive NMOSD	GCST006938	https://www.ebi.ac.uk/gwas/home
	AQP4-IgG-seronegative NMOSD	GCST006937	
	Combined NMOSD regardless of AQP4-IgG status	GCST005964	

- : Not feasible.

**Figure 2:**
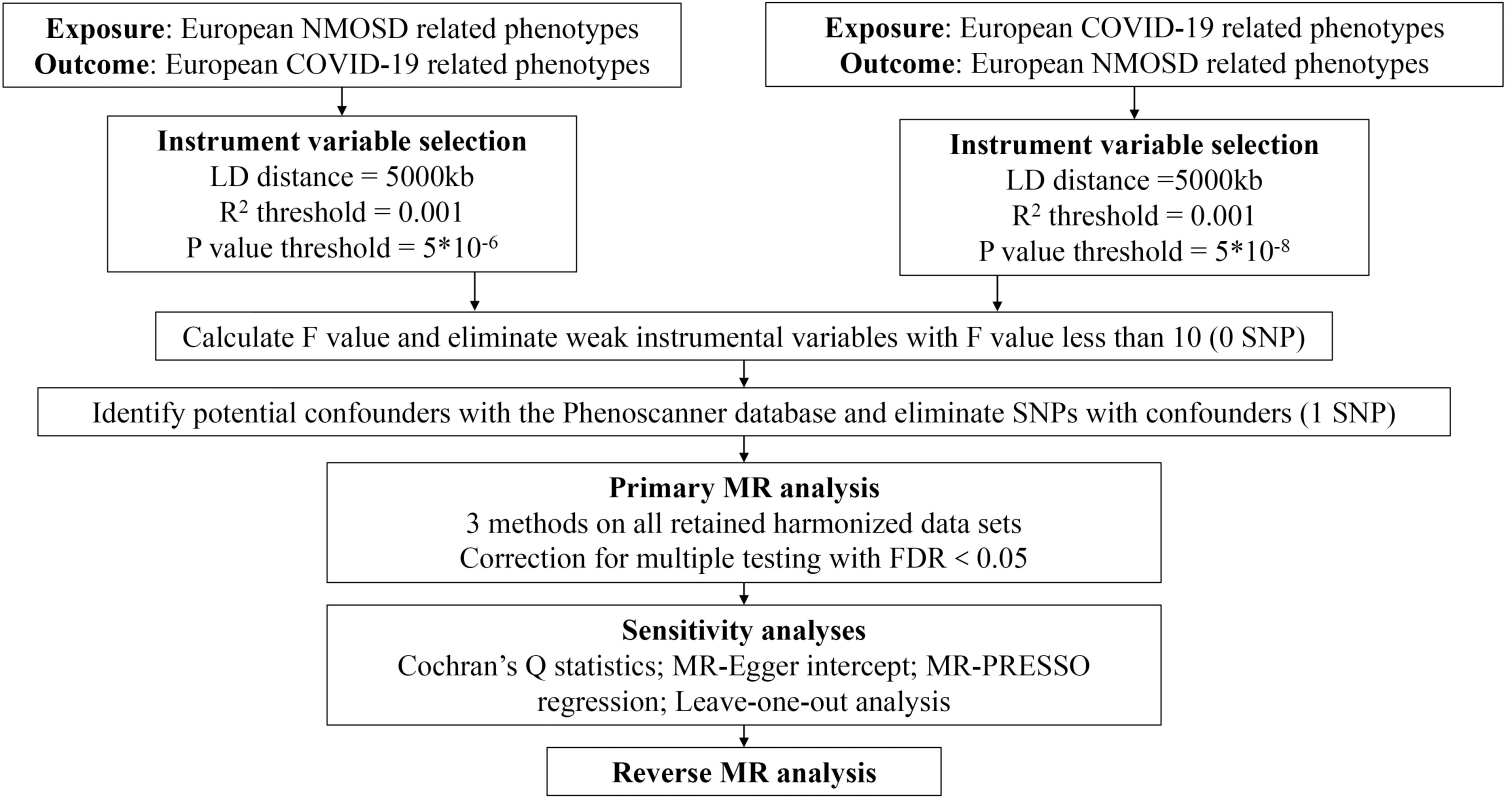
A complete workflow detailing the whole SNP selection procedure. * : multiplication.

To fulfill the initial assumption (30), we discovered genetic variations linked to COVID-19 exposure that satisfied the genome-wide significance threshold (p < 5 × 10^–8^). Due to the limited sample size of NMOSD, we established a P-value of 5 × 10^–6^ for instrumental variable selection in relation to exposure IVs. Afterwards, we removed SNPs that exhibited linkage disequilibrium (r^2^ < 0.001 within a 5000kb range) and retrieved the remaining SNPs from the outcome datasets (31). Additionally, we cross-referenced the Phenoscanner database (http://www.phenoscanner.medschl.cam.ac.uk/) to identify SNPs linked to the exposure that may be associated with confounding variables or outcomes (p < 5 × 10^–8^). To meet the assumptions of genetic instrumental variables being independent of the outcome and confounding factors, all relevant SNPs were manually excluded. In our findings, we utilized the MR-PRESSO technique to consider horizontal pleiotropy (32). Additionally, we employed the F-statistic to gauge the strength of the connection between instrumental variables and exposure, where an F-statistic below 10 signifies a feeble association (33). A complete workflow detailing the whole SNP selection procedure can be found in **Figure 2**.

### Statistical analysis

For our two-sample MR analyses, we utilized three distinct approaches: inverse variance weighted (IVW), MR-Egger, and weighted median. IVW was employed as the main MR analysis, while the other two approaches were utilized as supplementary analyses (34). To evaluate heterogeneity, we conducted Cochran’s Q test, whereas the pleiotropic effect was assessed by using the p-value for the intercept in MR-Egger regression. In addition, we employed MR-PRESSO for the detection and elimination of horizontal pleiotropic outliers (35). In addition, we performed leave-one-out sensitivity analysis to identify any possible influential SNPs, which aided in guaranteeing the dependability and consistency of the estimates for causal effects. The analyses were conducted utilizing TwoSampleMR (version 0.5.6) and MRPRESSO (version 1.0) packages within R (version 4.2.1).

## Results

**Figure 3:**
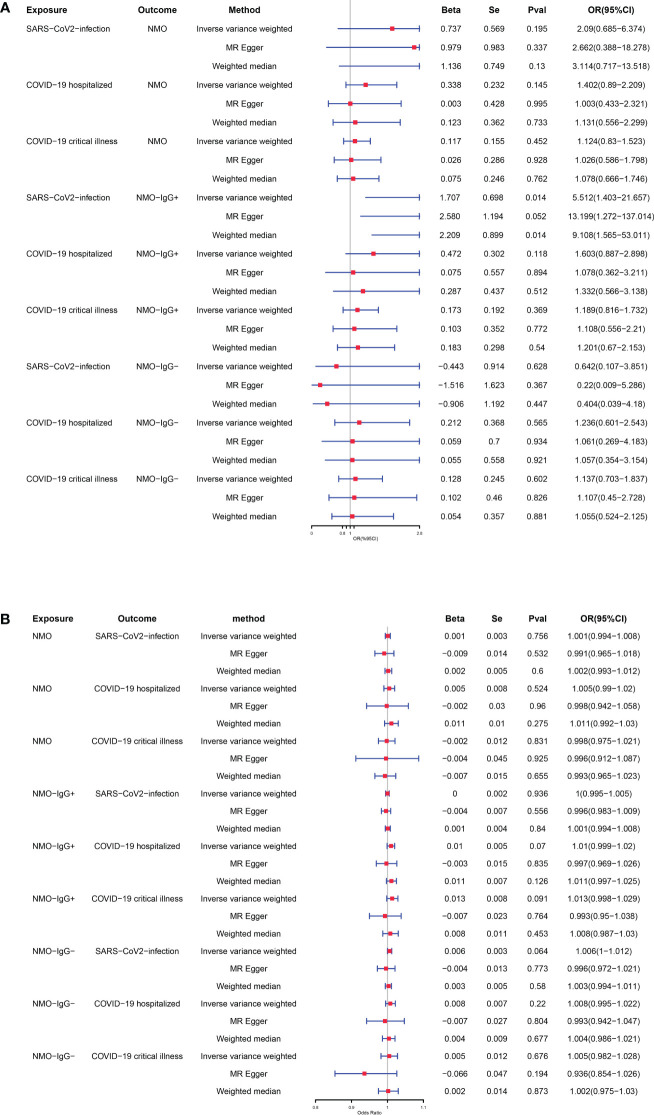
**(A)** Causal relationships between genetically predicted COVID-19 and the risk of NMOSD. **(B)** Causal relationships between genetically predicted NMOSD and the risk of COVID-19.

After applying strict exclusion criteria, the impact of NMOSD outcomes was evaluated by using COVID-19 as the exposure factor, and the selected SNPs were listed in **Supplementary Table 5**. NMOSD was employed as the variable of interest in reverse analyses to assess the influence of COVID-19 outcomes. **Supplementary Table 6** contained the chosen SNPs. The MR estimates obtained from various techniques employed to ascertain the causal impact of COVID-19 on NMOSD are illustrated in **Figure 3A**. According to the IVW approach, contracting SARS-CoV-2 raises the likelihood of developing NMO-IgG+ (odds ratio = 5.512, 95% confidence interval = 1.403-21.657, p-value = 0.014). According to Cochran’s Q test, there is no evidence of heterogeneity as all p-values were greater than 0.05. Based on the MR-Egger intercept, there was no indication of horizontal pleiotropy (p = 0.385, respectively). No influence was observed in the leave-one-out studies conducted for sensitivity analysis. The MR-PRESSO Global test indicates that there are no abnormal variations or horizontal pleiotropy in our findings (p = 0.945, respectively). **Supplementary Figures 1–4** includes the scatter plot, funnel plot, forest plot, and leave-one-out analysis plot that correspond to the given information. Moreover, there was no notable correlation observed between severe NMOSD, hospitalization due to NMOSD, and the likelihood of contracting COVID-19. The results of the **Supplementary Tables 7, 9, 11** provide information on the diversity, multiple effects, and MR-PRESSO Global test outcomes.

After conducting extensive Mendelian randomization analyses using various statistical techniques (including IVW, weighted median, and MR-Egger regression) and performing sensitivity analyses, we have concluded that there is no substantial proof to suggest a causal connection between any of the NMOSD phenotypes and the susceptibility to COVID-19, as depicted in **Figure 3B**. The **Supplementary Tables 8, 10, 12** provide the results for heterogeneity, pleiotropy, and MR-PRESSO Global test.

## Discussion

The connection between NMOSD and susceptibility to and severity of COVID-19 remains a subject of discussion. An area being examined is the possibility of individuals with NMOSD being more susceptible to acquiring COVID-19 or having an increased susceptibility to severe illness. Investigating another aspect involves examining the possibility of NMOSD patients in remission encountering a recurrence of NMOSD after contracting COVID-19.

Although certain scientists have proposed that individuals with NMOSD are more susceptible to acquiring COVID-19, numerous clinical investigations refute this assertion. Furthermore, although there are accounts of NMOSD relapses as a possible consequence of COVID-19 infection, the majority of research is constrained by inadequate sample sizes and factors that may cause confusion (1).

In order to fill this void, we carried out an extensive MR study with a large sample size to examine the causal impact of various levels of NMOSD on susceptibility, hospitalization, and severity of COVID-19. According to our research, individuals with a genetic inclination toward increased vulnerability to COVID-19 were found to have a greater likelihood of NMO-IgG+ (OR = 5.512, 95% CI = 1.403-21.657, P = 0.014).Hence, safeguarding the well-being of NMOSD patients is imperative in order to prevent the contraction of COVID-19. Examining GWAS data, this study pioneers the use of MR analysis to explore the correlation between NMOSD and COVID-19, providing a chance to speculate about the potential mechanism through which the virus affects the disease. Because all GWAS results has ruled out major confounders, the SNPs chosen to represent this phenotype are themselves unaffected by other characteristics of these patients.

Multiple research projects have indicated that the CNS can be infiltrated by the COVID-19 virus via either the indirect bloodstream or direct neural pathway, which may result in neurological complications (36). Recent evidence indicates that the SARS-CoV-2 Spike (S) protein demonstrates direct inflammatory and pro-coagulation properties. When combined with immune dysregulation leading to cytokine release syndrome (CRS), these effects may potentially contribute to the development of acute cerebrovascular or neuroinflammatory disorders (37). In a recent investigation utilizing a 3D microfluidic prototype, it was discovered that the S protein has the ability to disrupt the blood-brain barrier (BBB) and trigger an inflammatory reaction in brain endothelial cells (38). Furthermore, the loss of blood-brain barrier integrity in specific brain regions mediated by CRS may potentially lead to the activation of proinflammatory mediators by neural cells, thereby affecting brain function even after the acute infection has been resolved (37). In a recent study, a highly multiplexed spatial analysis was conducted on CNS tissue obtained from deceased COVID-19 patients to examine the cellular composition and immunological phenotype. The findings revealed a notable immune activation characterized by the presence of specific CD8 T-cell clusters affecting the vasculature and significant CD8 T-cell-microglial crosstalk in the parenchyma. This interaction often resulted in the formation of microglial nodules (39). During the remission stage of NMOSD, there is an equilibrium between the proinflammatory elements and the factors responsible for reducing inflammation (40). Hence, our speculation is that the equilibrium state of the central nervous system is disturbed by COVID-19 infection, resulting in the occurrence of sudden episodes or relapses. Multiple additional investigations have indicated that the virus exhibits cross-reactivity with immune cells and can imitate their function, resulting in bystander activation, epitope spreading, subclinical illness, and subsequent infiltration of inflammatory cells and AQP4-IgG antibodies in vulnerable individuals (41, 42). Hence, it has been suggested that SARS-CoV-2 infections may potentially initiate the development of NMO-IgG+. However, there is a continuing discussion about whether this connection is a result of the neurotropic characteristics of SARS-CoV-2 or a response triggered by the immune system, either immediately or with a delay (43, 44).

Certain patients who experience demyelinating symptoms after an infection exhibit genetic variants that are also present in traditional autoimmune diseases affecting the central nervous system. A recent investigation proposed that specific human leucocyte antigens (HLA)-DRB1 alleles associated with demyelinating diseases may increase the likelihood of developing CNS inflammatory demyelinating diseases (IDD) in individuals infected with the chikungunya virus (CHIKV) (45). Additionally, another study found that variations in the campylobacter gene determine both the reactivity of autoantibodies and the clinical manifestation of Guillain-Barré syndrome (GBS) (46). In their study, Martinelli-Boneschi, et al. explained the role of MS risk loci in post-infectious neurological syndromes (47).

On the other hand, there is a consensus that individuals with NMOSD are more prone to various infections as a result of utilizing immunosuppressive medications for disease management (48). However, limited research has been conducted on this subject matter. Our study did not find any proof of a causal connection between NMOSD and the vulnerability and seriousness of COVID-19. One study could be valid evidence to support our conclusion. During the early phases of the COVID-19 lockdown in China, research was carried out which revealed that individuals with MS or NMOSD did not face a higher chance of contracting COVID-19 (9). The reason for this is likely due to the fact that the selection of participants for this group was primarily based on patients from 10 MS centers located in 8 cities, which included Wuhan. The implementation of various rigorous precautions has helped safeguard these patients with neurological conditions, resulting in a comparatively low likelihood of COVID-19 transmission within their group. Our study yielded identical findings as mentioned earlier, indicating that the cumulative GWAS for COVID-19 could potentially offer more robust and persuasive outcomes. Our results were accurate due to the extensive size of the GWAS summary data.

Our study has a few limitations. To begin with, the specimen has a combination of European heritage. Additional research should be conducted on different racial or migrant populations in order to demonstrate the presence of a similar correlation. Furthermore, future research should include larger sample sizes and randomized controlled trials to validate our findings.

To summarize, our MR investigation revealed, for the initial occasion, that the presence of genetically predicted SARS-CoV-2 infection was identified as a contributing element to NMO-IgG+ relapses. Hence, it is imperative to safeguard individuals with NMOSD to avert the contraction of COVID-19. By doing so, the likelihood of repeated instances could be diminished, leading to a decrease in the estimated ratio of recurring risks.

## Data availability statement

The original contributions presented in the study are included in the article/**Supplementary Material**. Further inquiries can be directed to the corresponding author.

## Author contributions

SW: Writing – original draft. LW: Writing – review & editing. JW: Writing – original draft. MZ: Writing – review & editing.
