# Supplementary material for: Causal relationships between susceptibility and severity of COVID-19 and neuromyelitis optica spectrum disorder (NMOSD) in European population: a bidirectional Mendelian randomized study
Source: Front Immunol. 2023 Dec 4;14:1305650. doi: 10.3389/fimmu.2023.1305650 (PMC10726038; doi:10.3389/fimmu.2023.1305650)
Supplement: Supplementary file 1 [file DataSheet_1.docx]

Supplementary Table 1 Detailed information on SARS-CoV-2 infection gwas (122616 cases and 2475240 controls)

| **Name** | **n_cases** | **n_controls** |
| --- | --- | --- |
| ANCESTRY_Freeze_Four_EUR | 25353 | 113882 |
| CU_EUR | 508 | 2149 |
| Coronagenes_EUR | 181 | 734 |
| EXCEED_EUR | 236 | 1083 |
| EstBB_EUR | 4070 | 189406 |
| GENCOVID_EUR | 1697 | 2443 |
| GFG_EUR | 136 | 4978 |
| GHS_Freeze_145_EUR | 5276 | 108168 |
| Generation_Scotland_EUR | 1712 | 17525 |
| HUNT_EUR | 283 | 52072 |
| LGDB_EUR | 275 | 1313 |
| Lifelines_EUR | 895 | 26600 |
| MGI_EUR | 122 | 51458 |
| MOBA_EUR | 391 | 56038 |
| MVP_EUR | 11778 | 357198 |
| NTR_EUR | 228 | 5265 |
| PMBB_EUR | 60 | 9702 |
| SPGRX_EUR | 362 | 302 |
| Stanford_EUR | 169 | 190 |
| TOPMed_CHRIS10K_EUR | 92 | 2373 |
| TOPMed_Gardena_EUR | 452 | 458 |
| WGHS_EUR | 225 | 11887 |
| genomicsengland100kgp_EUR | 822 | 44369 |
| ABCD_EUR | 234 | 3135 |
| ALSPAC_G1_EUR | 523 | 4199 |
| BQC19_EUR | 1060 | 508 |
| BelCovid_EUR | 804 | 1477 |
| BioVU_EUR | 1592 | 68064 |
| BoSCO_EUR | 1168 | 984 |
| CCPM_EUR | 1496 | 24389 |
| DBDS_EUR | 5554 | 87127 |
| ERACORE_EUR | 210 | 1547 |
| FinnGen_FIN | 4814 | 337685 |
| GCAT_EUR | 710 | 4278 |
| Genotek_EUR | 6959 | 18720 |
| HOSTAGE_EUR | 3097 | 10327 |
| Helix_EUR | 1055 | 10960 |
| INMUNGEN_CoV2_EUR | 412 | 723 |
| PHBB_EUR | 1059 | 31558 |
| POLISH_COVID_WGS_EUR | 235 | 998 |
| SCOURGE_EUR | 9301 | 5455 |
| SINAI_COVID_EUR | 1329 | 8940 |
| TIKOCO_EUR | 572 | 3436 |
| UCLA_EUR | 846 | 20538 |
| UKBB_EUR | 14539 | 405992 |
| INTERVAL_EUR | 2098 | 39733 |
| Amsterdam_UMC_COVID_study_group_EUR | 108 | 1413 |
| DECODE_EUR | 89 | 274322 |
| idipaz24genetics_EUR | 106 | 75 |
| COMRI_EUR | 120 | 333 |
| Charite_EUR | 198 | 865 |
| FrenchCovid_EUR | 456 | 982 |
| Ioannina_EUR | 321 | 852 |
| Vanda_EUR | 58 | 900 |
| SweCovid_EUR | 181 | 3748 |
| genomicc_EUR | 5989 | 41384 |

Supplementary Table 2 Detailed information on hospitalized covid gwas (32519 cases and 2062805 controls)

| **Name** | **n_cases** | **n_controls** |
| --- | --- | --- |
| ANCESTRY_Freeze_Four_EUR | 1484 | 113882 |
| Amsterdam_UMC_COVID_study_group_EUR | 108 | 1413 |
| CU_EUR | 453 | 2149 |
| DECODE_EUR | 89 | 274322 |
| EstBB_EUR | 352 | 196455 |
| GENCOVID_EUR | 1287 | 2443 |
| GHS_Freeze_145_EUR | 773 | 108168 |
| Generation_Scotland_EUR | 554 | 18683 |
| LGDB_EUR | 57 | 1531 |
| MVP_EUR | 2417 | 366449 |
| SPGRX_EUR | 311 | 302 |
| idipaz24genetics_EUR | 106 | 75 |
| BQC19_EUR | 654 | 914 |
| BelCovid_EUR | 505 | 1477 |
| BioVU_EUR | 285 | 68064 |
| BoSCO_EUR | 425 | 984 |
| CCPM_EUR | 189 | 25696 |
| COMRI_EUR | 120 | 333 |
| Charite_EUR | 198 | 865 |
| FinnGen_FIN | 437 | 337685 |
| FrenchCovid_EUR | 456 | 982 |
| HOSTAGE_EUR | 3117 | 12483 |
| INMUNGEN_CoV2_EUR | 347 | 723 |
| Ioannina_EUR | 321 | 852 |
| PHBB_EUR | 318 | 31558 |
| POLISH_COVID_WGS_EUR | 359 | 871 |
| SCOURGE_EUR | 5934 | 8810 |
| UCLA_EUR | 150 | 21234 |
| UKBB_EUR | 3748 | 416783 |
| FHoGID_EUR | 470 | 280 |
| CGEN_EUR | 267 | 307 |
| Vanda_EUR | 58 | 900 |
| SweCovid_EUR | 181 | 3748 |
| genomicc_EUR | 5989 | 41384 |

Supplementary Table 3 Detailed information on very severe respiratory confirmed covid gwas

(13769 cases and 1072442 controls)

| **Name** | **n_cases** | **n_controls** |
| --- | --- | --- |
| ANCESTRY_Freeze_Four_EUR | 667 | 113882 |
| Amsterdam_UMC_COVID_study_group_EUR | 66 | 1413 |
| CU_EUR | 203 | 2149 |
| FinnGen_FIN | 190 | 278193 |
| GENCOVID_EUR | 1077 | 2443 |
| GHS_Freeze_145_EUR | 321 | 108168 |
| SPGRX_EUR | 101 | 302 |
| Vanda_EUR | 58 | 900 |
| idipaz24genetics_EUR | 59 | 75 |
| BQC19_EUR | 202 | 1366 |
| BelCovid_EUR | 297 | 1477 |
| BioVU_EUR | 117 | 68064 |
| BoSCO_EUR | 195 | 984 |
| Charite_EUR | 87 | 865 |
| HOSTAGE_EUR | 1850 | 12226 |
| INMUNGEN_CoV2_EUR | 81 | 723 |
| POLISH_COVID_WGS_EUR | 224 | 1005 |
| SCOURGE_EUR | 1124 | 13224 |
| SweCovid_EUR | 181 | 3748 |
| UKBB_EUR | 680 | 419851 |
| genomicc_EUR | 5989 | 41384 |

Supplementary Table 4 Study abbreviations of table 1-3

| **Full Name** | **Abbreviation** |
| --- | --- |
| 23andMe | 23ANDME |
| 24Genetics | idipaz24genetics |
| AArmenia_Covid-19hg | ArmCovid |
| ACCOuNT | ACCOuNT |
| Adolescent Brain and Cognitive development study | ABCD |
| Amsterdam UMC COVID study group | Amsterdam_UMC_COVID_study_group |
| Ancestry Freeze Four | ANCESTRY_Freeze_Four |
| Assessment of the influence of clinical, functional, immunological and genetic factors on the severity of the course of coronavirus infection with SARS-CoV-2 and Post Covid syndrome | KazCovid |
| Avon Longitudinal Study of Parents and Children (ALSPAC) | ALSPAC_G1 |
| Biobanque Quebec COVID19 | BQC19 |
| Bonn Study of COVID19 genetics | BoSCO |
| CCHC COVID-19 GAWS | CCHC |
| CGEn HostSeq - Canadian COVID-19 Human Host Genome Sequencing Databank | CGEN |
| CHOP CAG | CHOP_CAG |
| Columbia University COVID19 Biobank | CU |
| COMRI/Virology Study | COMRI |
| Coronagenes | Coronagenes |
| Covid19 Ioannina Biobank | Ioannina |
| COVID19-Host(a)ge | HOSTAGE |
| Covid19hg-CL | ChiCovid |
| deCODE | DECODE |
| Determining the Molecular Pathways and Genetic Predisposition of the Acute Inflammatory Process Caused by SARS-CoV-2 | SPGRX |
| Egypt hgCOVID hub | Egypt_hgCOVID_hub |
| EraCORE | ERACORE |
| Estonian Biobank | EstBB |
| EXCEED | EXCEED |
| FHoGID | FHoGID |
| FinnGen | FinnGen |
| French hgCOVID | FrenchCovid |
| Geisinger Health System | GHS_Freeze_145 |
| GEN-COVID, reCOVID | GENCOVID |
| Generation Scotland | Generation_Scotland |
| Genes & Health | GNH |
| Genes for Good | GFG |
| Genetic determinants of COVID-19 complications in the Brazilian population | BRACOVID |
| Genetic influences on severity of COVID-19 illness in Korea | Genetics_COVID19_Korea |
| Genetic modifiers for COVID-19 related illness | BelCovid |
| Genetics of COVID-related Manifestation | Corea |
| Genomes for Life | GCAT |
| Genomic epidemiology of SARS-Cov-2 and host genetics in Coronavirus Disease 2019 (COVID-19) | Stanford |
| genomiCC | genomicc |
| Genomics England | genomicsengland100kgp |
| Genotek COVID-19 study | Genotek |
| Helix Exome+ COVID-19 Phenotypes | Helix |
| Host genetic factors for COVID-19 severity and outcome in western Indian population | IND_GJ_COVID19 |
| Host genetic factors in COVID-19 patients in relation to disease susceptibility, disease severity and pharmacogenomics | thaicovid |
| Host Genetics in COVID cohorts of mixed ancestry from Mexico | MexCovid |
| HUNT | HUNT |
| Iran Covid | IranCovid |
| Japan Coronavirus Taskforce | JapanTaskForce |
| Jordan COVID-19 Host Genomics Initiative: (JCHGI) | JorCovid |
| Latvia COVID-19 research platform | LGDB |
| Lifelines | Lifelines |
| MexGen-COVID Initiative | MexGen-COVID |
| Michigan Genomics Initiative | MGI |
| Million Veterans Program | MVP |
| Mount Sinai Health System COVID-19 Genomics Initiative | SINAI_COVID |
| NCGM biobank | JSA-COVID19 |
| Netherlands Twin Register | NTR |
| Pa-COVID-19 | Charite |
| Partners Healthcare Biobank | PHBB |
| Penn Medicine Biobank | PMBB |
| Qatar Genome Program | QGP |
| SARS-CoV-2 and host genome sequencing | PakCovid |
| Saudi Human Genome Program | SaudiHumanGenomeProgram |
| Search for genomic markers predicting the severity of the response to COVID-19 | POLISH_COVID_WGS |
| Spanish COalition to Unlock Research on host GEnetics on COVID-19 (SCOURGE) | SCOURGE |
| The Colorado Center for Personalized Medicine | CCPM |
| The Danish Blood Donor Study | DBDS |
| The genetic predisposition to severe COVID-19 | SweCovid |
| The Norwegian Mother, Father and Child Cohort Study | MOBA |
| Tirschenreuth Study (TiKoco) | TIKOCO |
| TOPMed CHRIS | TOPMed_CHRIS10K |
| UCLA Precision Health COVID-19 Biobank | UCLA |
| UK Biobank | UKBB |
| UK Blood Donors Cohort | INTERVAL |
| Val Gardena | TOPMed_Gardena |
| Vanda COVID | Vanda |
| Vanderbilt Biobank | BioVU |
| Variability in immune response genes and severity of SARS-CoV-2 infection (INMUNGEN-CoV2 study) | INMUNGEN_CoV2 |
| Women's Health Genome Study | WGHS |

**Supplementary Table 5** Characteristics of the genetic instrument variables for NMO in the Mendelian randomization study at level P < 5 × 10^–6^

| **SNP** | **effect_allele** | **other_allele** | **beta.exposure** | **beta.outcome** | **chr.outcome** | **pos.outcome** | **pval.outcome** | **se.outcome** | **pval.exposure** | **se.exposure** | **F statistics** |
| --- | --- | --- | --- | --- | --- | --- | --- | --- | --- | --- | --- |
| **Exposure: NMO-IgG+ Outcome: COVID-19 critical illness** | | | | | | | | | | | |
| rs10049990 | G | A | 0.654 | 0.004 | 4 | 11764188 | 0.850 | 0.023 | 4.71E-06 | 0.143 | 20.952 |
| rs10199935 | G | T | -1.486 | -0.010 | 2 | 154677722 | 0.805 | 0.041 | 9.04E-07 | 0.303 | 24.122 |
| rs114255569 | T | A | -1.912 | -0.163 | 1 | 178073151 | 0.028 | 0.074 | 4.17E-06 | 0.415 | 21.183 |
| rs1150757 | G | A | -1.539 | -0.048 | 6 | 32061428 | 0.044 | 0.024 | 3.33E-16 | 0.189 | 66.600 |
| rs115956721 | G | A | -2.130 | 0.030 | 4 | 6612274 | 0.701 | 0.079 | 1.76E-06 | 0.446 | 22.840 |
| rs147488643 | C | T | -1.882 | 0.099 | 16 | 26124074 | 0.121 | 0.064 | 2.82E-06 | 0.402 | 21.933 |
| rs2017272 | T | A | -0.700 | 0.021 | 6 | 73486258 | 0.183 | 0.016 | 1.15E-06 | 0.144 | 23.657 |
| rs6920256 | G | A | -0.851 | -0.035 | 6 | 26537573 | 0.123 | 0.022 | 3.28E-06 | 0.183 | 21.647 |
| rs7158389 | T | A | -1.344 | -0.015 | 14 | 21472746 | 0.727 | 0.042 | 1.19E-06 | 0.277 | 23.587 |
| rs72736662 | T | A | 1.528 | -0.039 | 5 | 11837214 | 0.470 | 0.053 | 2.29E-06 | 0.323 | 22.336 |
| rs72831748 | G | A | -2.139 | -0.013 | 2 | 103096534 | 0.903 | 0.105 | 3.44E-06 | 0.461 | 21.552 |
| rs75746833 | C | T | 1.900 | -0.002 | 6 | 33635757 | 0.974 | 0.070 | 3.56E-06 | 0.410 | 21.489 |
| rs7604063 | G | A | -2.170 | 0.021 | 2 | 234236338 | 0.719 | 0.059 | 4.14E-06 | 0.471 | 21.201 |
| rs78235673 | C | T | -1.308 | -0.003 | 13 | 33527949 | 0.925 | 0.036 | 9.70E-07 | 0.267 | 23.987 |
| rs78727190 | G | A | -1.617 | -0.013 | 14 | 49429917 | 0.796 | 0.049 | 2.47E-06 | 0.343 | 22.191 |
| rs79295510 | C | A | 1.521 | 0.068 | 9 | 82744716 | 0.120 | 0.044 | 4.00E-06 | 0.330 | 21.263 |
| rs935411 | C | T | -0.938 | -0.013 | 2 | 238142909 | 0.624 | 0.026 | 4.47E-07 | 0.186 | 25.480 |
| **Exposure: NMO-IgG- Outcome: COVID-19 critical illness** | | | | | | | | | | | |
| rs112143562 | G | C | 1.360 | 0.033 | 2 | 26824003 | 0.301 | 0.032 | 1.09E-06 | 0.279 | 23.768 |
| rs113374594 | G | A | 2.058 | -0.200 | 11 | 77449236 | 0.044 | 0.099 | 3.63E-06 | 0.444 | 21.451 |
| rs116021398 | C | T | -1.353 | -0.004 | 2 | 103033027 | 0.903 | 0.033 | 2.56E-06 | 0.288 | 22.118 |
| rs138112111 | C | T | -2.274 | 0.016 | 6 | 96779376 | 0.829 | 0.072 | 2.03E-06 | 0.479 | 22.567 |
| rs140295126 | G | A | 2.144 | 0.012 | 6 | 136433948 | 0.834 | 0.059 | 3.46E-06 | 0.462 | 21.544 |
| rs142586613 | G | A | 1.815 | 0.072 | 14 | 68052218 | 0.213 | 0.058 | 4.68E-07 | 0.360 | 25.392 |
| rs17392655 | G | A | 1.457 | 0.001 | 12 | 12756141 | 0.980 | 0.039 | 2.42E-06 | 0.309 | 22.226 |
| rs17503897 | G | A | -1.821 | 0.029 | 11 | 82993232 | 0.705 | 0.077 | 1.89E-06 | 0.382 | 22.701 |
| rs17715143 | G | A | 1.052 | 0.082 | 18 | 40106832 | 0.017 | 0.034 | 5.40E-07 | 0.210 | 25.116 |
| rs7129918 | G | C | -1.467 | -0.042 | 11 | 39377880 | 0.370 | 0.047 | 3.74E-06 | 0.317 | 21.395 |
| rs74435765 | C | T | -1.447 | 0.035 | 1 | 185553812 | 0.417 | 0.044 | 8.76E-07 | 0.294 | 24.184 |
| rs74598405 | C | T | -2.098 | -0.040 | 2 | 177269237 | 0.718 | 0.110 | 2.78E-06 | 0.448 | 21.961 |
| **Exposure: NMO Outcome: COVID-19 critical illness** | | | | | | | | | | | |
| rs10199935 | G | T | -1.339 | -0.010 | 2 | 154677722 | 0.805 | 0.041 | 1.81E-07 | 0.257 | 27.229 |
| rs117785145 | G | T | -1.475 | 0.024 | 7 | 102768731 | 0.741 | 0.073 | 2.10E-06 | 0.311 | 22.506 |
| rs12353327 | C | T | -0.942 | 0.013 | 9 | 130134159 | 0.672 | 0.031 | 2.48E-06 | 0.200 | 22.181 |
| rs2716899 | C | T | -0.747 | -0.016 | 17 | 5706310 | 0.458 | 0.022 | 6.71E-07 | 0.150 | 24.696 |
| rs28383224 | G | A | -0.806 | 0.013 | 6 | 32615876 | 0.504 | 0.019 | 5.88E-12 | 0.117 | 47.371 |
| rs58134820 | G | C | 1.485 | -0.047 | 8 | 37815827 | 0.424 | 0.058 | 4.83E-06 | 0.325 | 20.904 |
| rs72831748 | G | A | -2.065 | -0.013 | 2 | 103096534 | 0.903 | 0.105 | 2.43E-07 | 0.400 | 26.661 |
| rs75504327 | G | A | 1.187 | -0.010 | 15 | 85581481 | 0.802 | 0.040 | 4.93E-06 | 0.260 | 20.864 |
| rs77496853 | C | T | -1.326 | -0.001 | 8 | 94064981 | 0.988 | 0.046 | 3.51E-07 | 0.260 | 25.949 |
| **Exposure: NMO-IgG+ Outcome: COVID-19 hospitalized** | | | | | | | | | | | |
| rs10049990 | G | A | 0.654 | 0.013 | 4 | 11764188 | 0.334 | 0.013 | 4.71E-06 | 0.143 | 20.952 |
| rs10199935 | G | T | -1.486 | -0.014 | 2 | 154677722 | 0.650 | 0.031 | 9.04E-07 | 0.303 | 24.122 |
| rs114255569 | T | A | -1.912 | -0.120 | 1 | 178073151 | 0.017 | 0.050 | 4.17E-06 | 0.415 | 21.183 |
| rs1150757 | G | A | -1.539 | -0.024 | 6 | 32061428 | 0.147 | 0.016 | 3.33E-16 | 0.189 | 66.600 |
| rs115956721 | G | A | -2.130 | -0.002 | 4 | 6612274 | 0.980 | 0.061 | 1.76E-06 | 0.446 | 22.840 |
| rs117979073 | G | A | 1.826 | 0.103 | 8 | 136807865 | 0.207 | 0.082 | 2.17E-06 | 0.385 | 22.438 |
| rs147488643 | C | T | -1.882 | 0.038 | 16 | 26124074 | 0.410 | 0.046 | 2.82E-06 | 0.402 | 21.933 |
| rs2017272 | T | A | -0.700 | -0.001 | 6 | 73486258 | 0.924 | 0.011 | 1.15E-06 | 0.144 | 23.657 |
| rs6920256 | G | A | -0.851 | -0.024 | 6 | 26537573 | 0.124 | 0.015 | 3.28E-06 | 0.183 | 21.647 |
| rs7158389 | T | A | -1.344 | -0.019 | 14 | 21472746 | 0.538 | 0.031 | 1.19E-06 | 0.277 | 23.587 |
| rs72736662 | T | A | 1.528 | -0.014 | 5 | 11837214 | 0.721 | 0.040 | 2.29E-06 | 0.323 | 22.336 |
| rs72831748 | G | A | -2.139 | -0.037 | 2 | 103096534 | 0.584 | 0.068 | 3.44E-06 | 0.461 | 21.552 |
| rs75746833 | C | T | 1.900 | 0.014 | 6 | 33635757 | 0.746 | 0.042 | 3.56E-06 | 0.410 | 21.489 |
| rs7604063 | G | A | -2.170 | 0.023 | 2 | 234236338 | 0.599 | 0.043 | 4.14E-06 | 0.471 | 21.201 |
| rs78235673 | C | T | -1.308 | 0.003 | 13 | 33527949 | 0.897 | 0.025 | 9.70E-07 | 0.267 | 23.987 |
| rs78727190 | G | A | -1.617 | 0.009 | 14 | 49429917 | 0.787 | 0.034 | 2.47E-06 | 0.343 | 22.191 |
| rs79295510 | C | A | 1.521 | 0.041 | 9 | 82744716 | 0.174 | 0.030 | 4.00E-06 | 0.330 | 21.263 |
| rs935411 | C | T | -0.938 | -0.001 | 2 | 238142909 | 0.976 | 0.018 | 4.47E-07 | 0.186 | 25.480 |
| **Exposure: NMO-IgG- Outcome: COVID-19 hospitalized** | | | | | | | | | | | |
| rs112143562 | G | C | 1.360 | 0.029 | 2 | 26824003 | 0.199 | 0.023 | 1.09E-06 | 0.279 | 23.768 |
| rs113374594 | G | A | 2.058 | 0.083 | 11 | 77449236 | 0.122 | 0.053 | 3.63E-06 | 0.444 | 21.451 |
| rs116021398 | C | T | -1.353 | -0.009 | 2 | 103033027 | 0.716 | 0.024 | 2.56E-06 | 0.288 | 22.118 |
| rs138112111 | C | T | -2.274 | 0.046 | 6 | 96779376 | 0.371 | 0.052 | 2.03E-06 | 0.479 | 22.567 |
| rs140295126 | G | A | 2.144 | -0.007 | 6 | 136433948 | 0.862 | 0.043 | 3.46E-06 | 0.462 | 21.544 |
| rs142586613 | G | A | 1.815 | -0.003 | 14 | 68052218 | 0.939 | 0.042 | 4.68E-07 | 0.360 | 25.392 |
| rs17392655 | G | A | 1.457 | 0.004 | 12 | 12756141 | 0.889 | 0.029 | 2.42E-06 | 0.309 | 22.226 |
| rs17503897 | G | A | -1.821 | -0.012 | 11 | 82993232 | 0.795 | 0.046 | 1.89E-06 | 0.382 | 22.701 |
| rs17715143 | G | A | 1.052 | 0.025 | 18 | 40106832 | 0.176 | 0.018 | 5.40E-07 | 0.210 | 25.116 |
| rs7129918 | G | C | -1.467 | -0.016 | 11 | 39377880 | 0.649 | 0.034 | 3.74E-06 | 0.317 | 21.395 |
| rs74435765 | C | T | -1.447 | 0.006 | 1 | 185553812 | 0.851 | 0.030 | 8.76E-07 | 0.294 | 24.184 |
| rs74598405 | C | T | -2.098 | -0.130 | 2 | 177269237 | 0.043 | 0.064 | 2.78E-06 | 0.448 | 21.961 |
| **Exposure: NMO Outcome: COVID-19 hospitalized** | | | | | | | | | | | |
| rs10199935 | G | T | -1.339 | -0.014 | 2 | 154677722 | 0.650 | 0.031 | 1.81E-07 | 0.257 | 27.229 |
| rs117785145 | G | T | -1.475 | 0.008 | 7 | 102768731 | 0.853 | 0.044 | 2.10E-06 | 0.311 | 22.506 |
| rs12353327 | C | T | -0.942 | 0.020 | 9 | 130134159 | 0.344 | 0.021 | 2.48E-06 | 0.200 | 22.181 |
| rs2716899 | C | T | -0.747 | -0.005 | 17 | 5706310 | 0.739 | 0.015 | 6.71E-07 | 0.150 | 24.696 |
| rs28383224 | G | A | -0.806 | -0.012 | 6 | 32615876 | 0.264 | 0.011 | 5.88E-12 | 0.117 | 47.371 |
| rs58134820 | G | C | 1.485 | -0.026 | 8 | 37815827 | 0.551 | 0.043 | 4.83E-06 | 0.325 | 20.904 |
| rs72831748 | G | A | -2.065 | -0.037 | 2 | 103096534 | 0.584 | 0.068 | 2.43E-07 | 0.400 | 26.661 |
| rs75504327 | G | A | 1.187 | -0.020 | 15 | 85581481 | 0.469 | 0.027 | 4.93E-06 | 0.260 | 20.864 |
| rs77496853 | C | T | -1.326 | -0.023 | 4 | 11765812 | 0.471 | 0.032 | 3.51E-07 | 0.260 | 25.949 |
| **Exposure: NMO-IgG+ Outcome: SARS-CoV2-infection** | | | | | | | | | | | |
| rs10049990 | G | A | 0.654 | -0.003 | 4 | 11765812 | 0.544 | 0.005 | 9.04E-07 | 0.143 | 20.952 |
| rs10199935 | G | T | -1.486 | 0.002 | 2 | 155534234 | 0.911 | 0.016 | 4.17E-06 | 0.303 | 24.122 |
| rs114255569 | T | A | -1.912 | -0.024 | 1 | 178042286 | 0.276 | 0.022 | 3.33E-16 | 0.415 | 21.183 |
| rs1150757 | G | A | -1.539 | -0.001 | 6 | 32029205 | 0.902 | 0.008 | 1.76E-06 | 0.189 | 66.600 |
| rs115956721 | G | A | -2.130 | 0.026 | 4 | 6614001 | 0.384 | 0.030 | 2.17E-06 | 0.446 | 22.840 |
| rs117979073 | G | A | 1.826 | 0.021 | 8 | 137820108 | 0.525 | 0.033 | 2.82E-06 | 0.385 | 22.438 |
| rs147488643 | C | T | -1.882 | -0.019 | 16 | 26135395 | 0.370 | 0.021 | 1.15E-06 | 0.402 | 21.933 |
| rs2017272 | T | A | -0.700 | 0.010 | 6 | 74195981 | 0.053 | 0.005 | 3.28E-06 | 0.144 | 23.657 |
| rs6920256 | G | A | -0.851 | -0.006 | 6 | 26537801 | 0.445 | 0.007 | 1.19E-06 | 0.183 | 21.647 |
| rs7158389 | T | A | -1.344 | 0.010 | 14 | 21940905 | 0.459 | 0.013 | 2.29E-06 | 0.277 | 23.587 |
| rs72736662 | T | A | 1.528 | -0.006 | 5 | 11837326 | 0.779 | 0.020 | 3.44E-06 | 0.323 | 22.336 |
| rs72831748 | G | A | -2.139 | -0.023 | 2 | 103712992 | 0.424 | 0.029 | 3.56E-06 | 0.461 | 21.552 |
| rs75746833 | C | T | 1.900 | -0.017 | 6 | 33603534 | 0.395 | 0.019 | 4.14E-06 | 0.410 | 21.489 |
| rs7604063 | G | A | -2.170 | 0.036 | 2 | 235144982 | 0.120 | 0.023 | 9.70E-07 | 0.471 | 21.201 |
| rs78235673 | C | T | -1.308 | -0.007 | 13 | 34102086 | 0.523 | 0.012 | 2.47E-06 | 0.267 | 23.987 |
| rs78727190 | G | A | -1.617 | 0.025 | 14 | 49896635 | 0.144 | 0.017 | 4.00E-06 | 0.343 | 22.191 |
| rs79295510 | C | A | 1.521 | 0.002 | 9 | 85359631 | 0.918 | 0.015 | 4.47E-07 | 0.330 | 21.263 |
| rs935411 | C | T | -0.938 | -0.012 | 2 | 239051550 | 0.139 | 0.008 | 9.04E-07 | 0.186 | 25.480 |
| **Exposure: NMO-IgG- Outcome: SARS-CoV2-infection** | | | | | | | | | | | |
| rs112143562 | G | C | 1.360 | -0.006 | 2 | 27046871 | 0.592 | 0.011 | 1.09E-06 | 0.279 | 23.768 |
| rs113374594 | G | A | 2.058 | 0.010 | 11 | 77160281 | 0.630 | 0.020 | 3.63E-06 | 0.444 | 21.451 |
| rs116021398 | C | T | -1.353 | -0.001 | 2 | 103649485 | 0.900 | 0.011 | 2.56E-06 | 0.288 | 22.118 |
| rs138112111 | C | T | -2.274 | 0.003 | 6 | 97227252 | 0.893 | 0.025 | 2.03E-06 | 0.479 | 22.567 |
| rs140295126 | G | A | 2.144 | 0.019 | 6 | 136755086 | 0.363 | 0.021 | 3.46E-06 | 0.462 | 21.544 |
| rs142586613 | G | A | 1.815 | -0.005 | 14 | 68518935 | 0.789 | 0.020 | 4.68E-07 | 0.360 | 25.392 |
| rs17392655 | G | A | 1.457 | -0.009 | 12 | 12909075 | 0.458 | 0.013 | 2.42E-06 | 0.309 | 22.226 |
| rs17503897 | G | A | -1.821 | -0.023 | 11 | 82704274 | 0.247 | 0.020 | 1.89E-06 | 0.382 | 22.701 |
| rs17715143 | G | A | 1.052 | 0.020 | 18 | 37686796 | 0.012 | 0.008 | 5.40E-07 | 0.210 | 25.116 |
| rs7129918 | G | C | -1.467 | 0.000 | 11 | 39399430 | 0.984 | 0.015 | 3.74E-06 | 0.317 | 21.395 |
| rs74435765 | C | T | -1.447 | 0.001 | 1 | 185522944 | 0.954 | 0.015 | 8.76E-07 | 0.294 | 24.184 |
| rs74598405 | C | T | -2.098 | -0.054 | 2 | 178133965 | 0.057 | 0.028 | 2.78E-06 | 0.448 | 21.961 |
| **Exposure: NMO Outcome: SARS-CoV2-infection** | | | | | | | | | | | |
| rs10199935 | G | T | -1.339 | 0.002 | 2 | 155534234 | 0.911 | 0.016 | 1.81E-07 | 0.257 | 27.229 |
| rs117785145 | G | T | -1.475 | 0.034 | 7 | 102409178 | 0.071 | 0.019 | 2.10E-06 | 0.311 | 22.506 |
| rs12353327 | C | T | -0.942 | -0.002 | 9 | 132896438 | 0.821 | 0.010 | 2.48E-06 | 0.200 | 22.181 |
| rs2716899 | C | T | -0.747 | -0.006 | 17 | 5609630 | 0.405 | 0.007 | 6.71E-07 | 0.150 | 24.696 |
| rs28383224 | G | A | -0.806 | -0.002 | 6 | 32583653 | 0.680 | 0.005 | 5.88E-12 | 0.117 | 47.371 |
| rs58134820 | G | C | 1.485 | 0.005 | 8 | 37673345 | 0.824 | 0.020 | 4.83E-06 | 0.325 | 20.904 |
| rs72831748 | G | A | -2.065 | -0.023 | 2 | 103712992 | 0.424 | 0.029 | 2.43E-07 | 0.400 | 26.661 |
| rs75504327 | G | A | 1.187 | -0.008 | 15 | 86124712 | 0.498 | 0.012 | 4.93E-06 | 0.260 | 20.864 |
| rs77496853 | C | T | -1.326 | -0.012 | 8 | 95077209 | 0.440 | 0.016 | 3.51E-07 | 0.260 | 25.949 |

**Supplementary Table 6** Characteristics of the genetic instrument variables for COVID-19 in the Mendelian randomization study at level P < 5 × 10^–8^

| **SNP** | **effect_allele** | **other_allele** | **beta.exposure** | **beta.outcome** | **chr.outcome** | **pos.outcome** | **pval.outcome** | **se.outcome** | **se.exposure** | **pval.exposure** | **F statistics** |
| --- | --- | --- | --- | --- | --- | --- | --- | --- | --- | --- | --- |
| **Exposure: SARS-CoV2-infection Outcome: NMO** | | | | | | | | | | | |
| rs10774673 | T | C | 0.029 | -0.087 | 12 | 113361158 | 0.437 | 0.111 | 0.005 | 2.14E-09 | 35.802 |
| rs1123573 | G | A | -0.026 | -0.035 | 2 | 60707588 | 0.753 | 0.112 | 0.005 | 3.16E-08 | 30.632 |
| rs11264339 | T | C | -0.035 | 0.015 | 1 | 155140648 | 0.893 | 0.111 | 0.005 | 1.62E-14 | 58.912 |
| rs12610495 | G | A | 0.050 | -0.084 | 19 | 4717672 | 0.477 | 0.118 | 0.005 | 1.80E-22 | 94.947 |
| rs142770866 | A | G | 0.047 | -0.215 | 19 | 10525372 | 0.319 | 0.216 | 0.008 | 2.91E-08 | 30.747 |
| rs184781326 | G | A | -0.069 | 0.085 | 14 | 29459234 | 0.744 | 0.259 | 0.012 | 3.42E-09 | 34.780 |
| rs2260685 | C | T | 0.033 | 0.108 | 3 | 195497743 | 0.324 | 0.110 | 0.005 | 1.01E-12 | 50.716 |
| rs2290859 | T | C | -0.051 | -0.018 | 3 | 101525625 | 0.878 | 0.117 | 0.005 | 1.32E-25 | 109.258 |
| rs2834158 | C | T | -0.041 | -0.055 | 21 | 34617213 | 0.623 | 0.112 | 0.005 | 8.51E-17 | 69.160 |
| rs35044562 | G | A | 0.128 | 0.090 | 3 | 45909024 | 0.647 | 0.196 | 0.008 | 1.64E-51 | 227.839 |
| rs554833 | C | T | -0.091 | -0.102 | 9 | 136147160 | 0.370 | 0.114 | 0.005 | 5.87E-81 | 363.880 |
| rs676314 | G | A | 0.028 | 0.073 | 19 | 50865535 | 0.512 | 0.111 | 0.005 | 1.02E-08 | 32.925 |
| rs679574 | G | C | -0.036 | 0.086 | 19 | 49206108 | 0.431 | 0.110 | 0.005 | 4.37E-15 | 61.248 |
| rs7118388 | G | A | 0.027 | 0.170 | 11 | 34454147 | 0.117 | 0.109 | 0.005 | 4.71E-09 | 34.480 |
| rs73062389 | A | G | 0.200 | 0.251 | 3 | 45835417 | 0.232 | 0.210 | 0.010 | 7.61E-92 | 411.440 |
| rs9264740 | T | C | -0.031 | 0.105 | 6 | 31244331 | 0.403 | 0.125 | 0.005 | 6.87E-09 | 33.622 |
| **Exposure: COVID-19 hospitalized Outcome: NMO** | | | | | | | | | | | |
| rs1123573 | G | A | -0.069 | -0.035 | 2 | 60707588 | 0.753 | 0.112 | 0.010 | 4.13E-11 | 43.763 |
| rs117169628 | A | G | 0.101 | 0.097 | 16 | 89262657 | 0.529 | 0.155 | 0.014 | 1.27E-13 | 54.350 |
| rs12585036 | T | C | 0.097 | 0.107 | 13 | 113535741 | 0.416 | 0.131 | 0.012 | 9.39E-17 | 69.492 |
| rs12610495 | G | A | 0.148 | -0.084 | 19 | 4717672 | 0.477 | 0.118 | 0.011 | 4.21E-41 | 181.025 |
| rs139589338 | G | A | 0.209 | 0.285 | 1 | 154826289 | 0.434 | 0.365 | 0.036 | 4.97E-09 | 34.273 |
| rs142770866 | A | G | 0.132 | -0.215 | 19 | 10525372 | 0.319 | 0.216 | 0.018 | 5.73E-14 | 56.250 |
| rs1498399 | G | A | 0.069 | -0.031 | 1 | 77947239 | 0.786 | 0.112 | 0.010 | 8.24E-12 | 46.402 |
| rs1634761 | T | C | -0.068 | -0.303 | 6 | 31274027 | 0.007 | 0.112 | 0.010 | 1.02E-12 | 50.784 |
| rs17279437 | A | G | -0.114 | 0.122 | 3 | 45814094 | 0.498 | 0.180 | 0.017 | 1.37E-11 | 46.046 |
| rs17412601 | C | T | -0.068 | -0.018 | 3 | 101499275 | 0.877 | 0.117 | 0.010 | 3.69E-11 | 43.971 |
| rs17885848 | T | C | 0.061 | 0.136 | 10 | 81316456 | 0.238 | 0.115 | 0.011 | 1.93E-08 | 31.422 |
| rs2102497 | C | T | 0.064 | -0.119 | 16 | 54255222 | 0.340 | 0.125 | 0.012 | 4.21E-08 | 30.297 |
| rs2326562 | T | C | 0.053 | 0.249 | 8 | 61428200 | 0.028 | 0.113 | 0.010 | 4.81E-08 | 29.722 |
| rs2897075 | T | C | 0.059 | -0.020 | 7 | 99630342 | 0.861 | 0.113 | 0.010 | 2.88E-09 | 35.201 |
| rs3014983 | T | C | -0.070 | -0.181 | 1 | 65460691 | 0.160 | 0.129 | 0.012 | 6.99E-09 | 33.543 |
| rs34536443 | C | G | 0.235 | -0.272 | 19 | 10463118 | 0.383 | 0.311 | 0.026 | 3.83E-20 | 84.929 |
| rs34712979 | A | G | -0.064 | 0.071 | 4 | 106819053 | 0.564 | 0.123 | 0.012 | 2.17E-08 | 31.263 |
| rs35705950 | T | G | -0.099 | -0.013 | 11 | 1241221 | 0.944 | 0.180 | 0.016 | 1.90E-10 | 40.713 |
| rs41264915 | G | A | -0.143 | -0.183 | 1 | 155167786 | 0.314 | 0.181 | 0.015 | 1.43E-20 | 86.224 |
| rs41435745 | C | G | 0.213 | 0.202 | 6 | 41490382 | 0.614 | 0.401 | 0.035 | 1.45E-09 | 36.616 |
| rs4475253 | G | A | 0.058 | 0.036 | 5 | 131776506 | 0.749 | 0.113 | 0.010 | 7.07E-09 | 33.205 |
| rs4767025 | T | C | 0.076 | -0.087 | 12 | 113358794 | 0.436 | 0.111 | 0.010 | 7.13E-14 | 55.879 |
| rs492602 | G | A | -0.055 | 0.089 | 19 | 49206417 | 0.414 | 0.110 | 0.010 | 7.81E-09 | 33.307 |
| rs5023077 | C | T | -0.066 | 0.003 | 12 | 133141973 | 0.981 | 0.107 | 0.010 | 3.79E-12 | 48.311 |
| rs61078946 | T | A | -0.090 | -0.087 | 16 | 75640517 | 0.626 | 0.179 | 0.016 | 3.12E-08 | 30.590 |
| rs61882275 | A | G | -0.092 | -0.197 | 11 | 34504292 | 0.084 | 0.114 | 0.010 | 2.30E-20 | 85.665 |
| rs63750417 | T | C | -0.092 | -0.007 | 17 | 44060775 | 0.956 | 0.132 | 0.012 | 1.37E-15 | 63.722 |
| rs676314 | G | A | 0.078 | 0.073 | 19 | 50865535 | 0.512 | 0.111 | 0.010 | 8.95E-15 | 59.641 |
| rs67959919 | A | G | 0.492 | 0.060 | 3 | 45871908 | 0.764 | 0.200 | 0.018 | 2.36E-173 | 790.413 |
| rs78314212 | T | C | 0.122 | 0.184 | 21 | 35312916 | 0.337 | 0.192 | 0.017 | 1.22E-12 | 50.311 |
| rs915823 | C | A | -0.069 | 0.057 | 21 | 42851454 | 0.659 | 0.130 | 0.013 | 4.33E-08 | 30.030 |
| rs9636867 | G | A | 0.128 | 0.042 | 21 | 34609944 | 0.710 | 0.114 | 0.010 | 2.05E-36 | 157.478 |
| **Exposure: COVID-19 critical illness Outcome: NMO** | | | | | | | | | | | |
| rs10850097 | T | C | 0.095 | -0.085 | 12 | 113361117 | 0.445 | 0.111 | 0.015 | 1.67E-10 | 40.823 |
| rs11208559 | G | C | 0.103 | 0.186 | 1 | 65435283 | 0.137 | 0.125 | 0.017 | 1.71E-09 | 36.281 |
| rs1123573 | G | A | -0.106 | -0.035 | 2 | 60707588 | 0.753 | 0.112 | 0.015 | 3.09E-12 | 48.632 |
| rs1128175 | G | A | -0.126 | 0.157 | 6 | 31150435 | 0.244 | 0.135 | 0.017 | 1.73E-13 | 54.294 |
| rs11614702 | A | G | 0.101 | 0.058 | 12 | 133058157 | 0.592 | 0.108 | 0.014 | 2.50E-13 | 53.565 |
| rs117169628 | A | G | 0.157 | 0.097 | 16 | 89262657 | 0.529 | 0.155 | 0.020 | 5.68E-15 | 61.011 |
| rs12534422 | T | C | 0.086 | -0.064 | 7 | 75263792 | 0.589 | 0.118 | 0.015 | 1.44E-08 | 32.136 |
| rs12585036 | T | C | 0.141 | 0.107 | 13 | 113535741 | 0.416 | 0.131 | 0.017 | 2.45E-16 | 67.202 |
| rs12610495 | G | A | 0.242 | -0.084 | 19 | 4717672 | 0.477 | 0.118 | 0.016 | 1.11E-51 | 228.766 |
| rs12614007 | A | G | 0.094 | 0.079 | 2 | 57316503 | 0.544 | 0.131 | 0.017 | 2.58E-08 | 31.003 |
| rs142770866 | A | G | 0.207 | -0.215 | 19 | 10525372 | 0.319 | 0.216 | 0.026 | 7.98E-16 | 64.875 |
| rs17279437 | A | G | -0.172 | 0.122 | 3 | 45814094 | 0.498 | 0.180 | 0.025 | 7.25E-12 | 46.958 |
| rs17713054 | A | G | 0.756 | 0.055 | 3 | 45859651 | 0.784 | 0.201 | 0.026 | 7.03E-186 | 845.467 |
| rs17885848 | T | C | 0.090 | 0.136 | 10 | 81316456 | 0.238 | 0.115 | 0.016 | 7.38E-09 | 33.432 |
| rs2897075 | T | C | 0.088 | -0.020 | 7 | 99630342 | 0.861 | 0.113 | 0.014 | 9.89E-10 | 37.346 |
| rs343320 | A | G | 0.154 | 0.157 | 3 | 146234909 | 0.447 | 0.206 | 0.028 | 2.14E-08 | 31.360 |
| rs34536443 | C | G | 0.381 | -0.272 | 19 | 10463118 | 0.383 | 0.311 | 0.037 | 3.01E-25 | 107.775 |
| rs34712979 | A | G | -0.110 | 0.071 | 4 | 106819053 | 0.564 | 0.123 | 0.017 | 9.76E-11 | 41.869 |
| rs35705950 | T | G | -0.164 | -0.013 | 11 | 1241221 | 0.944 | 0.180 | 0.023 | 6.34E-13 | 51.739 |
| rs368565 | T | C | 0.106 | -0.214 | 19 | 49201217 | 0.059 | 0.113 | 0.015 | 1.13E-12 | 50.610 |
| rs41264915 | G | A | -0.206 | -0.183 | 1 | 155167786 | 0.314 | 0.181 | 0.023 | 2.35E-19 | 80.921 |
| rs4608913 | G | A | -0.117 | -0.203 | 5 | 131760418 | 0.181 | 0.152 | 0.021 | 1.86E-08 | 31.641 |
| rs550057 | C | T | -0.117 | -0.102 | 9 | 136146597 | 0.399 | 0.121 | 0.016 | 1.86E-13 | 54.147 |
| rs568035 | T | C | 0.143 | 0.056 | 1 | 156110167 | 0.784 | 0.204 | 0.026 | 3.80E-08 | 30.250 |
| rs60132559 | T | C | 0.092 | 0.073 | 19 | 50867106 | 0.510 | 0.111 | 0.015 | 1.06E-09 | 37.210 |
| rs61882275 | A | G | -0.126 | -0.197 | 11 | 34504292 | 0.084 | 0.114 | 0.015 | 1.02E-17 | 73.469 |
| rs62056905 | G | A | -0.129 | -0.015 | 17 | 43782693 | 0.911 | 0.132 | 0.017 | 1.61E-14 | 58.960 |
| rs78258279 | G | A | 0.179 | 0.142 | 21 | 35327265 | 0.452 | 0.188 | 0.025 | 8.07E-13 | 51.266 |
| rs9305744 | A | G | -0.099 | -0.005 | 21 | 42842988 | 0.968 | 0.124 | 0.017 | 7.78E-09 | 33.330 |
| rs9636867 | G | A | 0.184 | 0.042 | 21 | 34609944 | 0.710 | 0.114 | 0.015 | 3.72E-34 | 148.485 |
| **Exposure: SARS-CoV2-infection Outcome: NMO-IgG-** | | | | | | | | | | | |
| rs10774673 | T | C | 0.029 | -0.210 | 12 | 113361158 | 0.211 | 0.168 | 0.005 | 2.14E-09 | 35.802 |
| rs1123573 | G | A | -0.026 | -0.111 | 2 | 60707588 | 0.517 | 0.171 | 0.005 | 3.16E-08 | 30.632 |
| rs11264339 | T | C | -0.035 | 0.044 | 1 | 155140648 | 0.794 | 0.168 | 0.005 | 1.62E-14 | 58.912 |
| rs12610495 | G | A | 0.050 | 0.014 | 19 | 4717672 | 0.937 | 0.180 | 0.005 | 1.80E-22 | 94.947 |
| rs142770866 | A | G | 0.047 | -0.140 | 19 | 10525372 | 0.705 | 0.370 | 0.008 | 2.91E-08 | 30.747 |
| rs184781326 | G | A | -0.069 | 0.139 | 14 | 29459234 | 0.713 | 0.377 | 0.012 | 3.42E-09 | 34.780 |
| rs2260685 | C | T | 0.033 | 0.098 | 3 | 195497743 | 0.556 | 0.167 | 0.005 | 1.01E-12 | 50.716 |
| rs2290859 | T | C | -0.051 | 0.083 | 3 | 101525625 | 0.635 | 0.175 | 0.005 | 1.32E-25 | 109.258 |
| rs2834158 | C | T | -0.041 | 0.059 | 21 | 34617213 | 0.735 | 0.174 | 0.005 | 8.51E-17 | 69.160 |
| rs35044562 | G | A | 0.128 | -0.028 | 3 | 45909024 | 0.933 | 0.328 | 0.008 | 1.64E-51 | 227.839 |
| rs554833 | C | T | -0.091 | 0.128 | 9 | 136147160 | 0.476 | 0.179 | 0.005 | 5.87E-81 | 363.880 |
| rs676314 | G | A | 0.028 | 0.182 | 19 | 50865535 | 0.264 | 0.163 | 0.005 | 1.02E-08 | 32.925 |
| rs679574 | G | C | -0.036 | 0.198 | 19 | 49206108 | 0.237 | 0.168 | 0.005 | 4.37E-15 | 61.248 |
| rs7118388 | G | A | 0.027 | 0.261 | 11 | 34454147 | 0.122 | 0.169 | 0.005 | 4.71E-09 | 34.480 |
| rs73062389 | A | G | 0.200 | -0.092 | 3 | 45835417 | 0.799 | 0.360 | 0.010 | 7.61E-92 | 411.440 |
| rs9264740 | T | C | -0.031 | 0.078 | 6 | 31244331 | 0.684 | 0.190 | 0.005 | 6.87E-09 | 33.622 |
| **Exposure: COVID-19 hospitalized Outcome: NMO-IgG-** | | | | | | | | | | | |
| rs1123573 | G | A | -0.069 | -0.111 | 2 | 60707588 | 0.517 | 0.171 | 0.010 | 4.13E-11 | 43.763 |
| rs117169628 | A | G | 0.101 | -0.223 | 16 | 89262657 | 0.396 | 0.262 | 0.014 | 1.27E-13 | 54.350 |
| rs12585036 | T | C | 0.097 | 0.080 | 13 | 113535741 | 0.689 | 0.199 | 0.012 | 9.39E-17 | 69.492 |
| rs12610495 | G | A | 0.148 | 0.014 | 19 | 4717672 | 0.937 | 0.180 | 0.011 | 4.21E-41 | 181.025 |
| rs142770866 | A | G | 0.132 | -0.140 | 19 | 10525372 | 0.705 | 0.370 | 0.018 | 5.73E-14 | 56.250 |
| rs1498399 | G | A | 0.069 | -0.073 | 1 | 77947239 | 0.671 | 0.171 | 0.010 | 8.24E-12 | 46.402 |
| rs1634761 | T | C | -0.068 | -0.051 | 6 | 31274027 | 0.757 | 0.166 | 0.010 | 1.02E-12 | 50.784 |
| rs17279437 | A | G | -0.114 | 0.018 | 3 | 45814094 | 0.949 | 0.280 | 0.017 | 1.37E-11 | 46.046 |
| rs17412601 | C | T | -0.068 | 0.085 | 3 | 101499275 | 0.628 | 0.176 | 0.010 | 3.69E-11 | 43.971 |
| rs17885848 | T | C | 0.061 | -0.008 | 10 | 81316456 | 0.964 | 0.176 | 0.011 | 1.93E-08 | 31.422 |
| rs2102497 | C | T | 0.064 | -0.132 | 16 | 54255222 | 0.486 | 0.189 | 0.012 | 4.21E-08 | 30.297 |
| rs2326562 | T | C | 0.053 | 0.172 | 8 | 61428200 | 0.313 | 0.170 | 0.010 | 4.81E-08 | 29.722 |
| rs2897075 | T | C | 0.059 | -0.017 | 7 | 99630342 | 0.920 | 0.169 | 0.010 | 2.88E-09 | 35.201 |
| rs3014983 | T | C | -0.070 | -0.136 | 1 | 65460691 | 0.482 | 0.194 | 0.012 | 6.99E-09 | 33.543 |
| rs34536443 | C | G | 0.235 | 0.246 | 19 | 10463118 | 0.526 | 0.388 | 0.026 | 3.83E-20 | 84.929 |
| rs34712979 | A | G | -0.064 | -0.191 | 4 | 106819053 | 0.341 | 0.201 | 0.012 | 2.17E-08 | 31.263 |
| rs35705950 | T | G | -0.099 | -0.328 | 11 | 1241221 | 0.280 | 0.303 | 0.016 | 1.90E-10 | 40.713 |
| rs41264915 | G | A | -0.143 | -0.252 | 1 | 155167786 | 0.375 | 0.284 | 0.015 | 1.43E-20 | 86.224 |
| rs41435745 | C | G | 0.213 | 0.404 | 6 | 41490382 | 0.473 | 0.562 | 0.035 | 1.45E-09 | 36.616 |
| rs4475253 | G | A | 0.058 | 0.030 | 5 | 131776506 | 0.862 | 0.175 | 0.010 | 7.07E-09 | 33.205 |
| rs4767025 | T | C | 0.076 | -0.210 | 12 | 113358794 | 0.210 | 0.168 | 0.010 | 7.13E-14 | 55.879 |
| rs492602 | G | A | -0.055 | 0.201 | 19 | 49206417 | 0.232 | 0.168 | 0.010 | 7.81E-09 | 33.307 |
| rs5023077 | C | T | -0.066 | -0.135 | 12 | 133141973 | 0.413 | 0.165 | 0.010 | 3.79E-12 | 48.311 |
| rs61078946 | T | A | -0.090 | 0.043 | 16 | 75640517 | 0.871 | 0.263 | 0.016 | 3.12E-08 | 30.590 |
| rs61882275 | A | G | -0.092 | -0.321 | 11 | 34504292 | 0.074 | 0.180 | 0.010 | 2.30E-20 | 85.665 |
| rs63750417 | T | C | -0.092 | 0.046 | 17 | 44060775 | 0.817 | 0.198 | 0.012 | 1.37E-15 | 63.722 |
| rs676314 | G | A | 0.078 | 0.182 | 19 | 50865535 | 0.264 | 0.163 | 0.010 | 8.95E-15 | 59.641 |
| rs67959919 | A | G | 0.492 | 0.026 | 3 | 45871908 | 0.938 | 0.328 | 0.018 | 2.36E-173 | 790.413 |
| rs78314212 | T | C | 0.122 | 0.002 | 21 | 35312916 | 0.995 | 0.319 | 0.017 | 1.22E-12 | 50.311 |
| rs915823 | C | A | -0.069 | 0.082 | 21 | 42851454 | 0.675 | 0.196 | 0.013 | 4.33E-08 | 30.030 |
| rs9636867 | G | A | 0.128 | -0.087 | 21 | 34609944 | 0.622 | 0.177 | 0.010 | 2.05E-36 | 157.478 |
| **Exposure: COVID-19 critical illness Outcome: NMO-IgG-** | | | | | | | | | | | |
| rs10850097 | T | C | 0.095 | -0.198 | 12 | 113361117 | 0.239 | 0.168 | 0.015 | 1.67E-10 | 40.823 |
| rs11208559 | G | C | 0.103 | 0.214 | 1 | 65435283 | 0.249 | 0.185 | 0.017 | 1.71E-09 | 36.281 |
| rs1123573 | G | A | -0.106 | -0.111 | 2 | 60707588 | 0.517 | 0.171 | 0.015 | 3.09E-12 | 48.632 |
| rs1128175 | G | A | -0.126 | 0.093 | 6 | 31150435 | 0.643 | 0.200 | 0.017 | 1.73E-13 | 54.294 |
| rs11614702 | A | G | 0.101 | 0.142 | 12 | 133058157 | 0.385 | 0.163 | 0.014 | 2.50E-13 | 53.565 |
| rs117169628 | A | G | 0.157 | -0.223 | 16 | 89262657 | 0.396 | 0.262 | 0.020 | 5.68E-15 | 61.011 |
| rs12534422 | T | C | 0.086 | -0.187 | 7 | 75263792 | 0.306 | 0.182 | 0.015 | 1.44E-08 | 32.136 |
| rs12585036 | T | C | 0.141 | 0.080 | 13 | 113535741 | 0.689 | 0.199 | 0.017 | 2.45E-16 | 67.202 |
| rs12610495 | G | A | 0.242 | 0.014 | 19 | 4717672 | 0.937 | 0.180 | 0.016 | 1.11E-51 | 228.766 |
| rs12614007 | A | G | 0.094 | -0.009 | 2 | 57316503 | 0.963 | 0.198 | 0.017 | 2.58E-08 | 31.003 |
| rs142770866 | A | G | 0.207 | -0.140 | 19 | 10525372 | 0.705 | 0.370 | 0.026 | 7.98E-16 | 64.875 |
| rs17279437 | A | G | -0.172 | 0.018 | 3 | 45814094 | 0.949 | 0.280 | 0.025 | 7.25E-12 | 46.958 |
| rs17713054 | A | G | 0.756 | 0.040 | 3 | 45859651 | 0.902 | 0.328 | 0.026 | 7.03E-186 | 845.467 |
| rs17885848 | T | C | 0.090 | -0.008 | 10 | 81316456 | 0.964 | 0.176 | 0.016 | 7.38E-09 | 33.432 |
| rs2897075 | T | C | 0.088 | -0.017 | 7 | 99630342 | 0.920 | 0.169 | 0.014 | 9.89E-10 | 37.346 |
| rs343320 | A | G | 0.154 | 0.141 | 3 | 146234909 | 0.644 | 0.305 | 0.028 | 2.14E-08 | 31.360 |
| rs34536443 | C | G | 0.381 | 0.246 | 19 | 10463118 | 0.526 | 0.388 | 0.037 | 3.01E-25 | 107.775 |
| rs34712979 | A | G | -0.110 | -0.191 | 4 | 106819053 | 0.341 | 0.201 | 0.017 | 9.76E-11 | 41.869 |
| rs35705950 | T | G | -0.164 | -0.328 | 11 | 1241221 | 0.280 | 0.303 | 0.023 | 6.34E-13 | 51.739 |
| rs368565 | T | C | 0.106 | -0.177 | 19 | 49201217 | 0.305 | 0.172 | 0.015 | 1.13E-12 | 50.610 |
| rs41264915 | G | A | -0.206 | -0.252 | 1 | 155167786 | 0.375 | 0.284 | 0.023 | 2.35E-19 | 80.921 |
| rs4608913 | G | A | -0.117 | -0.342 | 5 | 131760418 | 0.120 | 0.220 | 0.021 | 1.86E-08 | 31.641 |
| rs550057 | C | T | -0.117 | 0.118 | 9 | 136146597 | 0.548 | 0.196 | 0.016 | 1.86E-13 | 54.147 |
| rs568035 | T | C | 0.143 | -0.016 | 1 | 156110167 | 0.960 | 0.319 | 0.026 | 3.80E-08 | 30.250 |
| rs60132559 | T | C | 0.092 | 0.187 | 19 | 50867106 | 0.251 | 0.163 | 0.015 | 1.06E-09 | 37.210 |
| rs61882275 | A | G | -0.126 | -0.321 | 11 | 34504292 | 0.074 | 0.180 | 0.015 | 1.02E-17 | 73.469 |
| rs62056905 | G | A | -0.129 | 0.042 | 17 | 43782693 | 0.831 | 0.199 | 0.017 | 1.61E-14 | 58.960 |
| rs78258279 | G | A | 0.179 | 0.082 | 21 | 35327265 | 0.781 | 0.295 | 0.025 | 8.07E-13 | 51.266 |
| rs9305744 | A | G | -0.099 | 0.132 | 21 | 42842988 | 0.472 | 0.183 | 0.017 | 7.78E-09 | 33.330 |
| rs9636867 | G | A | 0.184 | -0.087 | 21 | 34609944 | 0.622 | 0.177 | 0.015 | 3.72E-34 | 148.485 |
| **Exposure: SARS-CoV2-infection Outcome: NMO-IgG+** | | | | | | | | | | | |
| rs10774673 | T | C | 0.029 | 0.000 | 12 | 113361158 | 0.999 | 0.133 | 0.005 | 2.14E-09 | 35.802 |
| rs1123573 | G | A | -0.026 | 0.005 | 2 | 60707588 | 0.971 | 0.139 | 0.005 | 3.16E-08 | 30.632 |
| rs11264339 | T | C | -0.035 | 0.002 | 1 | 155140648 | 0.987 | 0.138 | 0.005 | 1.62E-14 | 58.912 |
| rs12610495 | G | A | 0.050 | -0.147 | 19 | 4717672 | 0.324 | 0.149 | 0.005 | 1.80E-22 | 94.947 |
| rs142770866 | A | G | 0.047 | -0.065 | 19 | 10525372 | 0.803 | 0.259 | 0.008 | 2.91E-08 | 30.747 |
| rs184781326 | G | A | -0.069 | 0.031 | 14 | 29459234 | 0.926 | 0.332 | 0.012 | 3.42E-09 | 34.780 |
| rs2260685 | C | T | 0.033 | 0.120 | 3 | 195497743 | 0.385 | 0.138 | 0.005 | 1.01E-12 | 50.716 |
| rs2290859 | T | C | -0.051 | -0.099 | 3 | 101525625 | 0.504 | 0.149 | 0.005 | 1.32E-25 | 109.258 |
| rs2834158 | C | T | -0.041 | -0.130 | 21 | 34617213 | 0.351 | 0.139 | 0.005 | 8.51E-17 | 69.160 |
| rs35044562 | G | A | 0.128 | 0.223 | 3 | 45909024 | 0.347 | 0.237 | 0.008 | 1.64E-51 | 227.839 |
| rs554833 | C | T | -0.091 | -0.250 | 9 | 136147160 | 0.073 | 0.139 | 0.005 | 5.87E-81 | 363.880 |
| rs679574 | G | C | -0.036 | 0.011 | 19 | 49206108 | 0.934 | 0.137 | 0.005 | 4.37E-15 | 61.248 |
| rs7118388 | G | A | 0.027 | 0.109 | 11 | 34454147 | 0.417 | 0.135 | 0.005 | 4.71E-09 | 34.480 |
| rs73062389 | A | G | 0.200 | 0.457 | 3 | 45835417 | 0.063 | 0.245 | 0.010 | 7.61E-92 | 411.440 |
| rs9264740 | T | C | -0.031 | 0.129 | 6 | 31244331 | 0.412 | 0.157 | 0.005 | 6.87E-09 | 33.622 |
| **Exposure: COVID-19 hospitalized Outcome: NMO-IgG+** | | | | | | | | | | | |
| rs1123573 | G | A | -0.069 | 0.005 | 2 | 60707588 | 0.971 | 0.139 | 0.010 | 4.13E-11 | 43.763 |
| rs117169628 | A | G | 0.101 | 0.280 | 16 | 89262657 | 0.128 | 0.184 | 0.014 | 1.27E-13 | 54.350 |
| rs12585036 | T | C | 0.097 | 0.122 | 13 | 113535741 | 0.456 | 0.163 | 0.012 | 9.39E-17 | 69.492 |
| rs12610495 | G | A | 0.148 | -0.147 | 19 | 4717672 | 0.324 | 0.149 | 0.011 | 4.21E-41 | 181.025 |
| rs142770866 | A | G | 0.132 | -0.065 | 19 | 10525372 | 0.803 | 0.259 | 0.018 | 5.73E-14 | 56.250 |
| rs1498399 | G | A | 0.069 | 0.001 | 1 | 77947239 | 0.993 | 0.146 | 0.010 | 8.24E-12 | 46.402 |
| rs1634761 | T | C | -0.068 | -0.476 | 6 | 31274027 | 0.001 | 0.144 | 0.010 | 1.02E-12 | 50.784 |
| rs17279437 | A | G | -0.114 | 0.182 | 3 | 45814094 | 0.413 | 0.221 | 0.017 | 1.37E-11 | 46.046 |
| rs17412601 | C | T | -0.068 | -0.101 | 3 | 101499275 | 0.499 | 0.149 | 0.010 | 3.69E-11 | 43.971 |
| rs17885848 | T | C | 0.061 | 0.230 | 10 | 81316456 | 0.110 | 0.144 | 0.011 | 1.93E-08 | 31.422 |
| rs2102497 | C | T | 0.064 | -0.102 | 16 | 54255222 | 0.511 | 0.155 | 0.012 | 4.21E-08 | 30.297 |
| rs2326562 | T | C | 0.053 | 0.293 | 8 | 61428200 | 0.039 | 0.142 | 0.010 | 4.81E-08 | 29.722 |
| rs2897075 | T | C | 0.059 | -0.025 | 7 | 99630342 | 0.858 | 0.142 | 0.010 | 2.88E-09 | 35.201 |
| rs3014983 | T | C | -0.070 | -0.207 | 1 | 65460691 | 0.198 | 0.161 | 0.012 | 6.99E-09 | 33.543 |
| rs34536443 | C | G | 0.235 | -0.713 | 19 | 10463118 | 0.151 | 0.496 | 0.026 | 3.83E-20 | 84.929 |
| rs34712979 | A | G | -0.064 | 0.213 | 4 | 106819053 | 0.150 | 0.148 | 0.012 | 2.17E-08 | 31.263 |
| rs35705950 | T | G | -0.099 | 0.161 | 11 | 1241221 | 0.454 | 0.215 | 0.016 | 1.90E-10 | 40.713 |
| rs41264915 | G | A | -0.143 | -0.125 | 1 | 155167786 | 0.576 | 0.223 | 0.015 | 1.43E-20 | 86.224 |
| rs41435745 | C | G | 0.213 | 0.088 | 6 | 41490382 | 0.866 | 0.517 | 0.035 | 1.45E-09 | 36.616 |
| rs4475253 | G | A | 0.058 | 0.040 | 5 | 131776506 | 0.775 | 0.141 | 0.010 | 7.07E-09 | 33.205 |
| rs4767025 | T | C | 0.076 | 0.000 | 12 | 113358794 | 0.998 | 0.150 | 0.010 | 7.13E-14 | 55.879 |
| rs492602 | G | A | -0.055 | 0.015 | 19 | 49206417 | 0.914 | 0.138 | 0.010 | 7.81E-09 | 33.307 |
| rs5023077 | C | T | -0.066 | 0.091 | 12 | 133141973 | 0.503 | 0.136 | 0.010 | 3.79E-12 | 48.311 |
| rs61078946 | T | A | -0.090 | -0.153 | 16 | 75640517 | 0.510 | 0.232 | 0.016 | 3.12E-08 | 30.590 |
| rs61882275 | A | G | -0.092 | -0.114 | 11 | 34504292 | 0.417 | 0.141 | 0.010 | 2.30E-20 | 85.665 |
| rs63750417 | T | C | -0.092 | -0.009 | 17 | 44060775 | 0.956 | 0.171 | 0.012 | 1.37E-15 | 63.722 |
| rs676314 | G | A | 0.078 | -0.002 | 19 | 50865535 | 0.988 | 0.144 | 0.010 | 8.95E-15 | 59.641 |
| rs67959919 | A | G | 0.492 | 0.153 | 3 | 45871908 | 0.530 | 0.244 | 0.018 | 2.36E-173 | 790.413 |
| rs78314212 | T | C | 0.122 | 0.299 | 21 | 35312916 | 0.199 | 0.233 | 0.017 | 1.22E-12 | 50.311 |
| rs915823 | C | A | -0.069 | 0.038 | 21 | 42851454 | 0.813 | 0.163 | 0.013 | 4.33E-08 | 30.030 |
| rs9636867 | G | A | 0.128 | 0.126 | 21 | 34609944 | 0.367 | 0.140 | 0.010 | 2.05E-36 | 157.478 |
| **Exposure: COVID-19 critical illness Outcome: NMO-IgG+** | | | | | | | | | | | |
| rs10850097 | T | C | 0.095 | -0.006 | 12 | 113361117 | 0.969 | 0.141 | 0.015 | 1.67E-10 | 40.823 |
| rs11208559 | G | C | 0.103 | 0.163 | 1 | 65435283 | 0.298 | 0.157 | 0.017 | 1.71E-09 | 36.281 |
| rs1123573 | G | A | -0.106 | 0.005 | 2 | 60707588 | 0.971 | 0.139 | 0.015 | 3.09E-12 | 48.632 |
| rs1128175 | G | A | -0.126 | 0.209 | 6 | 31150435 | 0.223 | 0.172 | 0.017 | 1.73E-13 | 54.294 |
| rs11614702 | A | G | 0.101 | 0.008 | 12 | 133058157 | 0.952 | 0.135 | 0.014 | 2.50E-13 | 53.565 |
| rs117169628 | A | G | 0.157 | 0.280 | 16 | 89262657 | 0.128 | 0.184 | 0.020 | 5.68E-15 | 61.011 |
| rs12534422 | T | C | 0.086 | 0.035 | 7 | 75263792 | 0.812 | 0.147 | 0.015 | 1.44E-08 | 32.136 |
| rs12585036 | T | C | 0.141 | 0.122 | 13 | 113535741 | 0.456 | 0.163 | 0.017 | 2.45E-16 | 67.202 |
| rs12610495 | G | A | 0.242 | -0.147 | 19 | 4717672 | 0.324 | 0.149 | 0.016 | 1.11E-51 | 228.766 |
| rs12614007 | A | G | 0.094 | 0.121 | 2 | 57316503 | 0.467 | 0.166 | 0.017 | 2.58E-08 | 31.003 |
| rs142770866 | A | G | 0.207 | -0.065 | 19 | 10525372 | 0.803 | 0.259 | 0.026 | 7.98E-16 | 64.875 |
| rs17279437 | A | G | -0.172 | 0.182 | 3 | 45814094 | 0.413 | 0.221 | 0.025 | 7.25E-12 | 46.958 |
| rs17713054 | A | G | 0.756 | 0.140 | 3 | 45859651 | 0.571 | 0.247 | 0.026 | 7.03E-186 | 845.467 |
| rs17885848 | T | C | 0.090 | 0.230 | 10 | 81316456 | 0.110 | 0.144 | 0.016 | 7.38E-09 | 33.432 |
| rs2897075 | T | C | 0.088 | -0.025 | 7 | 99630342 | 0.858 | 0.142 | 0.014 | 9.89E-10 | 37.346 |
| rs343320 | A | G | 0.154 | 0.222 | 3 | 146234909 | 0.408 | 0.268 | 0.028 | 2.14E-08 | 31.360 |
| rs34536443 | C | G | 0.381 | -0.713 | 19 | 10463118 | 0.151 | 0.496 | 0.037 | 3.01E-25 | 107.775 |
| rs34712979 | A | G | -0.110 | 0.213 | 4 | 106819053 | 0.150 | 0.148 | 0.017 | 9.76E-11 | 41.869 |
| rs35705950 | T | G | -0.164 | 0.161 | 11 | 1241221 | 0.454 | 0.215 | 0.023 | 6.34E-13 | 51.739 |
| rs368565 | T | C | 0.106 | -0.233 | 19 | 49201217 | 0.102 | 0.142 | 0.015 | 1.13E-12 | 50.610 |
| rs41264915 | G | A | -0.206 | -0.125 | 1 | 155167786 | 0.576 | 0.223 | 0.023 | 2.35E-19 | 80.921 |
| rs4608913 | G | A | -0.117 | -0.097 | 5 | 131760418 | 0.621 | 0.196 | 0.021 | 1.86E-08 | 31.641 |
| rs550057 | C | T | -0.117 | -0.233 | 9 | 136146597 | 0.110 | 0.146 | 0.016 | 1.86E-13 | 54.147 |
| rs568035 | T | C | 0.143 | 0.087 | 1 | 156110167 | 0.730 | 0.252 | 0.026 | 3.80E-08 | 30.250 |
| rs60132559 | T | C | 0.092 | -0.006 | 19 | 50867106 | 0.966 | 0.142 | 0.015 | 1.06E-09 | 37.210 |
| rs61882275 | A | G | -0.126 | -0.114 | 11 | 34504292 | 0.417 | 0.141 | 0.015 | 1.02E-17 | 73.469 |
| rs62056905 | G | A | -0.129 | -0.019 | 17 | 43782693 | 0.910 | 0.171 | 0.017 | 1.61E-14 | 58.960 |
| rs78258279 | G | A | 0.179 | 0.194 | 21 | 35327265 | 0.405 | 0.234 | 0.025 | 8.07E-13 | 51.266 |
| rs9305744 | A | G | -0.099 | -0.094 | 21 | 42842988 | 0.556 | 0.159 | 0.017 | 7.78E-09 | 33.330 |
| rs9636867 | G | A | 0.184 | 0.126 | 21 | 34609944 | 0.367 | 0.140 | 0.015 | 3.72E-34 | 148.485 |

Supplementary Table 7 MR Egger intercept analysis of the association between COVID-19 and NMO risk.

| Exposure | Outcome | Egger_intercept | Se | Pval |
| --- | --- | --- | --- | --- |
| SARS-CoV2-infection | NMO | -0.017 | 0.056 | 0.768 |
| COVID-19 hospitalized | NMO | 0.041 | 0.044 | 0.360 |
| COVID-19 critical illness | NMO | 0.017 | 0.046 | 0.708 |
| SARS-CoV2-infection | NMO-IgG- | 0.071 | 0.089 | 0.438 |
| COVID-19 hospitalized | NMO-IgG- | 0.018 | 0.070 | 0.798 |
| COVID-19 critical illness | NMO-IgG- | 0.005 | 0.073 | 0.948 |
| SARS-CoV2-infection | NMO-IgG+ | -0.065 | 0.072 | 0.385 |
| COVID-19 hospitalized | NMO-IgG+ | 0.049 | 0.058 | 0.402 |
| COVID-19 critical illness | NMO-IgG+ | 0.014 | 0.057 | 0.814 |

Supplementary Table 8 MR Egger intercept analysis of the association between NMO and COVID-19 risk.

| Exposure | Outcome | Egger_intercept | Se | Pval |
| --- | --- | --- | --- | --- |
| NMO | SARS-CoV2-infection | 0.010 | 0.013 | 0.472 |
| NMO | COVID-19 hospitalized | 0.006 | 0.028 | 0.832 |
| NMO | COVID-19 critical illness | 0.002 | 0.044 | 0.966 |
| NMO-IgG- | SARS-CoV2-infection | 0.015 | 0.019 | 0.453 |
| NMO-IgG- | COVID-19 hospitalized | 0.024 | 0.041 | 0.576 |
| NMO-IgG- | COVID-19 critical illness | 0.113 | 0.072 | 0.156 |
| NMO-IgG+ | SARS-CoV2-infection | 0.005 | 0.008 | 0.506 |
| NMO-IgG+ | COVID-19 hospitalized | 0.017 | 0.018 | 0.370 |
| NMO-IgG+ | COVID-19 critical illness | 0.027 | 0.029 | 0.364 |

Supplementary Table 9 Heterogeneity analysis of COVID-19 and NMO risk.

| Exposure | Outcome | Method | Q | Q_df | Q_pval |
| --- | --- | --- | --- | --- | --- |
| SARS-CoV2-infection | NMO | MR Egger | 7.824 | 13 | 0.855 |
|  |  | Inverse variance weighted | 7.915 | 14 | 0.894 |
| COVID-19 hospitalized | NMO | MR Egger | 24.788 | 27 | 0.586 |
|  |  | Inverse variance weighted | 25.656 | 28 | 0.592 |
| COVID-19 critical illness | NMO | MR Egger | 18.997 | 26 | 0.837 |
|  |  | Inverse variance weighted | 19.140 | 27 | 0.865 |
| SARS-CoV2-infection | NMO-IgG- | MR Egger | 6.530 | 13 | 0.925 |
|  |  | Inverse variance weighted | 7.170 | 14 | 0.928 |
| COVID-19 hospitalized | NMO-IgG- | MR Egger | 15.048 | 26 | 0.956 |
|  |  | Inverse variance weighted | 15.115 | 27 | 0.968 |
| COVID-19 critical illness | NMO-IgG- | MR Egger | 16.904 | 26 | 0.912 |
|  |  | Inverse variance weighted | 16.908 | 27 | 0.933 |
| SARS-CoV2-infection | NMO-IgG+ | MR Egger | 5.220 | 12 | 0.950 |
|  |  | Inverse variance weighted | 6.033 | 13 | 0.945 |
| COVID-19 hospitalized | NMO-IgG+ | MR Egger | 28.587 | 26 | 0.330 |
|  |  | Inverse variance weighted | 29.385 | 27 | 0.342 |
| COVID-19 critical illness | NMO-IgG+ | MR Egger | 20.448 | 26 | 0.770 |
|  |  | Inverse variance weighted | 20.504 | 27 | 0.809 |

Supplementary Table 10 Heterogeneity analysis of NMO and COVID-19 risk.

| Exposure | Outcome | Method | Q | Q_df | Q_pval |
| --- | --- | --- | --- | --- | --- |
| NMO | SARS-CoV2-infection | MR Egger | 5.189 | 6 | 0.520 |
|  |  | Inverse variance weighted | 5.777 | 7 | 0.566 |
| NMO | COVID-19 hospitalized | MR Egger | 3.383 | 6 | 0.759 |
|  |  | Inverse variance weighted | 3.433 | 7 | 0.842 |
| NMO | COVID-19 critical illness | MR Egger | 1.378 | 6 | 0.967 |
|  |  | Inverse variance weighted | 1.380 | 7 | 0.986 |
| NMO-IgG- | SARS-CoV2-infection | MR Egger | 8.711 | 8 | 0.367 |
|  |  | Inverse variance weighted | 9.388 | 9 | 0.402 |
| NMO-IgG- | COVID-19 hospitalized | MR Egger | 7.566 | 8 | 0.477 |
|  |  | Inverse variance weighted | 7.905 | 9 | 0.544 |
| NMO-IgG- | COVID-19 critical illness | MR Egger | 9.267 | 8 | 0.320 |
|  |  | Inverse variance weighted | 12.112 | 9 | 0.207 |
| NMO-IgG+ | SARS-CoV2-infection | MR Egger | 10.995 | 12 | 0.529 |
|  |  | Inverse variance weighted | 11.464 | 13 | 0.572 |
| NMO-IgG+ | COVID-19 hospitalized | MR Egger | 6.365 | 12 | 0.897 |
|  |  | Inverse variance weighted | 7.233 | 13 | 0.890 |
| NMO-IgG+ | COVID-19 critical illness | MR Egger | 8.204 | 11 | 0.695 |
|  |  | Inverse variance weighted | 9.101 | 12 | 0.694 |

Supplementary Table 11 MR PRESSO Global test of the association between NMO and COVID-19 risk.

| Exposure | Outcome | Global.Test.RSSobs | Global.Test.Pvalue |
| --- | --- | --- | --- |
| NMO | SARS-CoV2-infection | 6.857 | 0.719 |
| NMO | COVID-19 hospitalized | 5.408 | 0.859 |
| NMO | COVID-19 critical illness | 2.508 | 0.98 |
| NMO-IgG- | SARS-CoV2-infection | 13.517 | 0.436 |
| NMO-IgG- | COVID-19 hospitalized | 9.709 | 0.701 |
| NMO-IgG- | COVID-19 critical illness | 14.940 | 0.359 |
| NMO-IgG+ | SARS-CoV2-infection | 18.878 | 0.469 |
| NMO-IgG+ | COVID-19 hospitalized | 13.364 | 0.827 |
| NMO-IgG+ | COVID-19 critical illness | 19.974 | 0.362 |

Supplementary Table 12 MR PRESSO Global test of the association between COVID-19 and NMO risk.

| Exposure | Outcome | Global.Test.RSSobs | Global.Test.Pvalue |
| --- | --- | --- | --- |
| SARS-CoV2-infection | NMO | 9.815 | 0.913 |
| COVID-19 hospitalized | NMO | 28.571 | 0.696 |
| COVID-19 critical illness | NMO | 23.240 | 0.84 |
| SARS-CoV2-infection | NMO-IgG- | 8.841 | 0.949 |
| COVID-19 hospitalized | NMO-IgG- | 16.668 | 0.988 |
| COVID-19 critical illness | NMO-IgG- | 19.167 | 0.954 |
| SARS-CoV2-infection | NMO-IgG+ | 7.318 | 0.963 |
| COVID-19 hospitalized | NMO-IgG+ | 33.866 | 0.401 |
| COVID-19 critical illness | NMO-IgG+ | 25.128 | 0.755 |


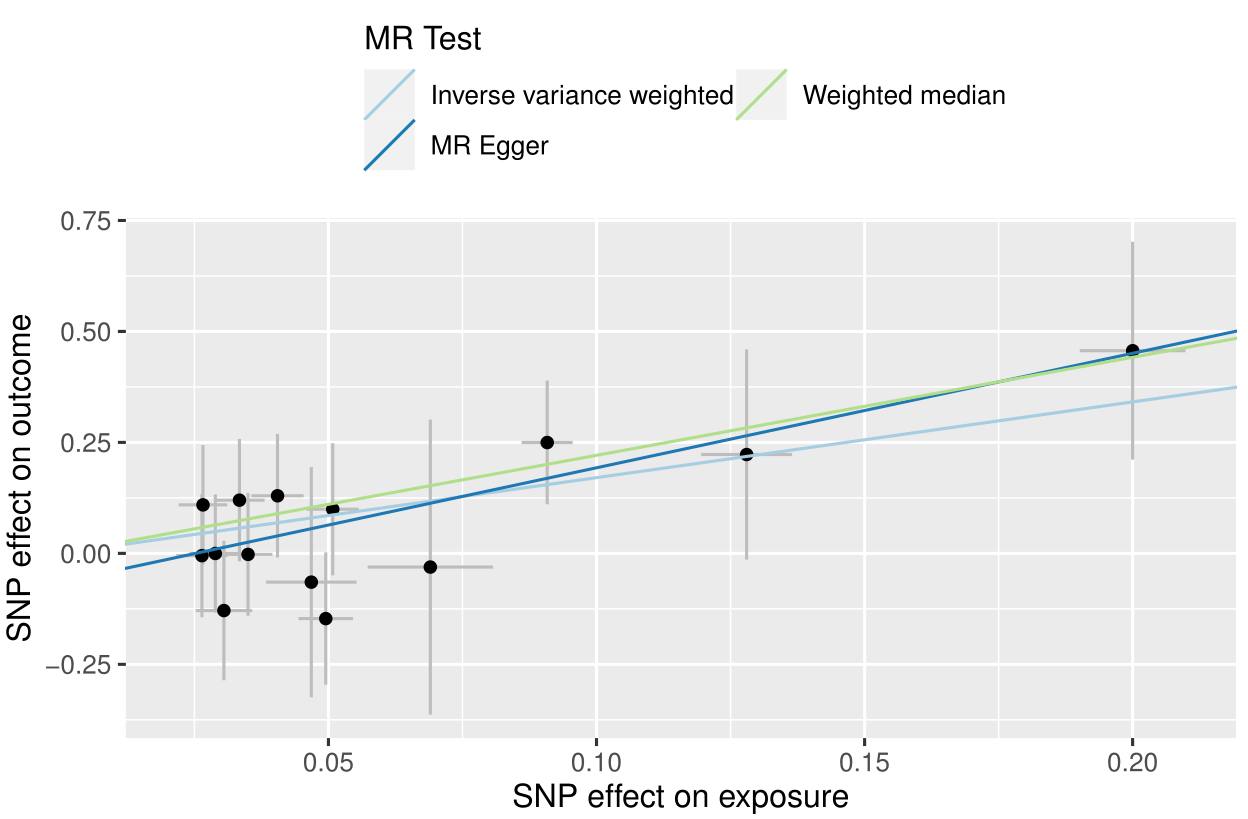


Supplementary Figure 1 The scatter plot for the association between SARS-CoV2-infection and NMO-IgG+ risk


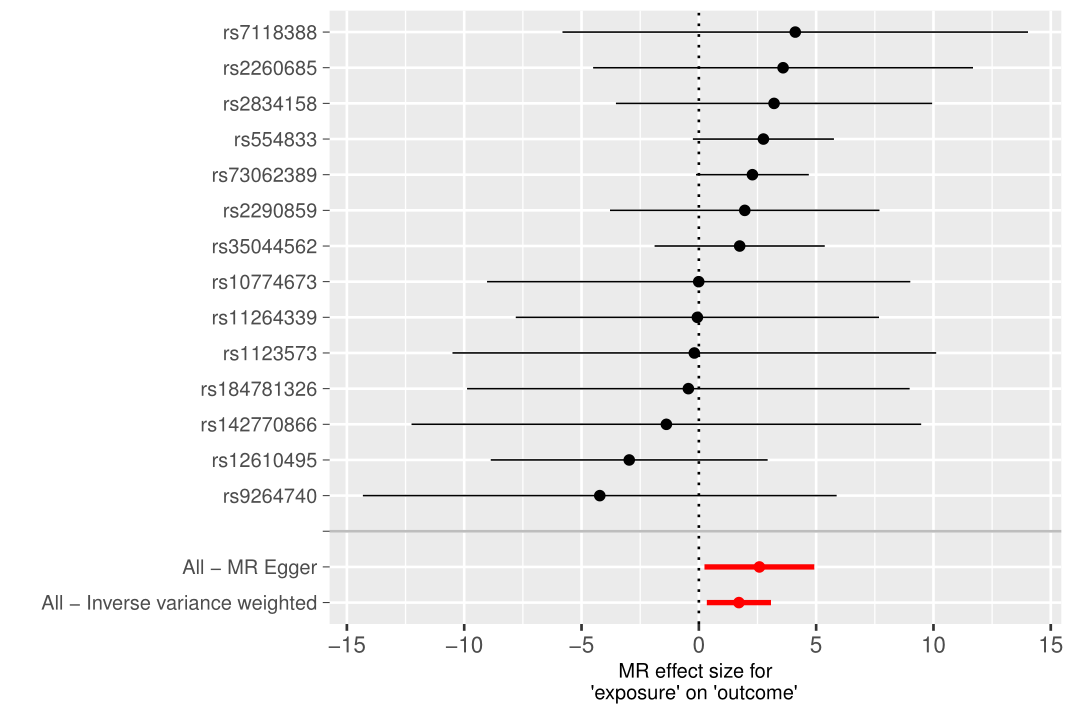


Supplementary Figure 2 The forest plot for the association between SARS-CoV2-infection and NMO-IgG+ risk


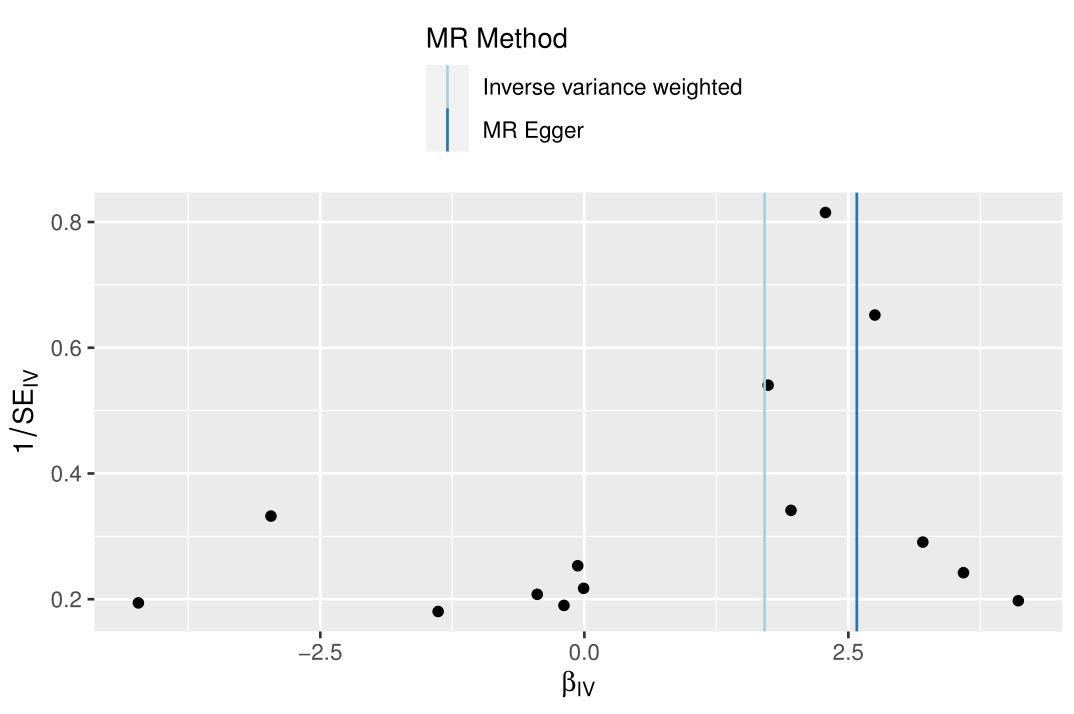


Supplementary Figure 3 The funnel plot for the association between SARS-CoV2-infection and NMO-IgG+ risk


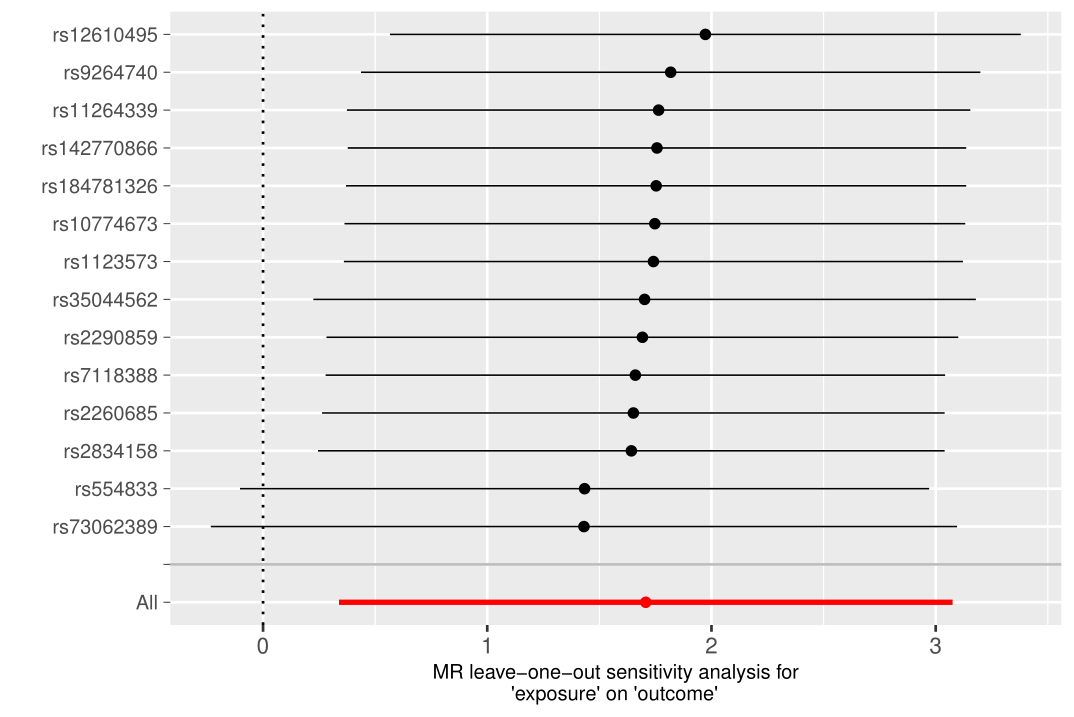


Supplementary Figure 4 MR leave−one−out sensitivity analysis for SARS-CoV2-infection and NMO-IgG+ risk
